# Supplementary material for: Plasticity of Lgr5-Negative Cancer Cells Drives Metastasis in Colorectal Cancer
Source: Cell Stem Cell. 2020 Apr 2;26(4):569–578.e7. doi: 10.1016/j.stem.2020.02.008 (PMC7118369; doi:10.1016/j.stem.2020.02.008)
Supplement: Document S1. Figures S1–S4 [file mmc1.pdf]

Cell Stem Cell, Volume *26*

Supplemental Information

Plasticity of Lgr5-Negative Cancer Cells

Drives Metastasis in Colorectal Cancer

Arianna Fumagalli, Koen C. Oost, Lennart Kester, Jessica Morgner, Laura Bornes, Lotte Bruens, Lisa Spaargaren, Maria Azkanaz, Tim Schelfhorst, Evelyne Beerling, Maria C. Heinz, Daniel Postrach, Danielle Seinstra, Anieta M. Sieuwerts, John W.M. Martens, Stefan van der Elst, Martijn van Baalen, Debajit Bhowmick, Nienke Vrisekoop, Saskia I.J. Ellenbroek, Saskia J.E. Suijkerbuijk, Hugo J. Snippert, and Jacco van Rheenen

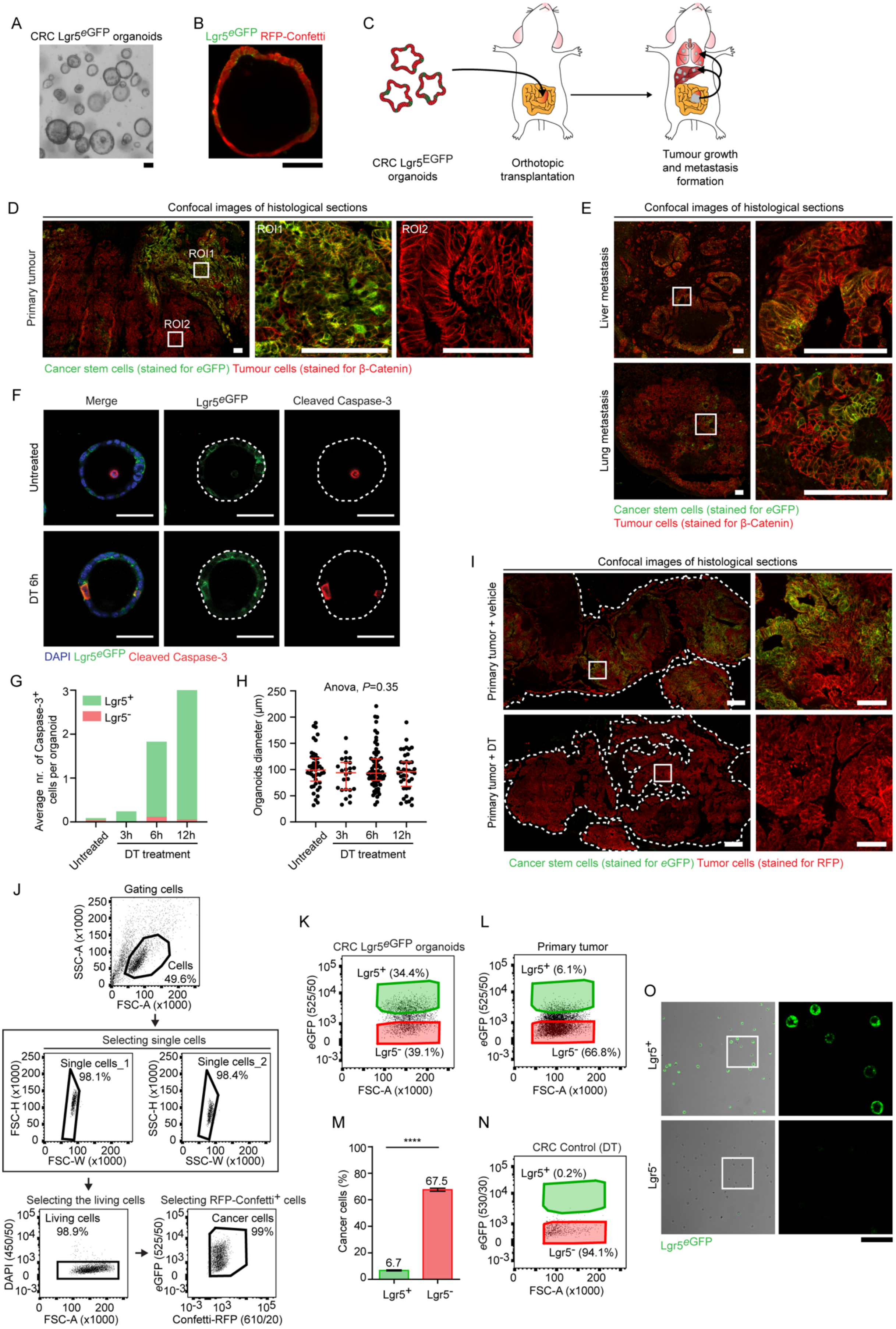

A
CRC Lgr5eGFP organoids
B
Lgr5eGFP RFP-Confetti
C
CRC Lgr5EGFP organoids
Orthotopic transplantation
Tumour growth and metastasis formation
D
Confocal images of histological sections
Primary tumour
ROI1
ROI2
Cancer stem cells (stained for eGFP) Tumour cells (stained for β-Catenin)
E
Confocal images of histological sections
Liver metastasis
Lung metastasis
Cancer stem cells (stained for eGFP)
Tumour cells (stained for β-Catenin)
F
Merge
Lgr5eGFP
Cleaved Caspase-3
Untreated
DT 6h
DAPI Lgr5eGFP Cleaved Caspase-3
G
Average nr. of Caspase-3+ cells per organoid
Lgr5+
Lgr5-
Untreated
3h
6h
12h
DT treatment
H
Anova, P=0.35
Organoids diameter (µm)
Untreated
3h
6h
12h
DT treatment
I
Confocal images of histological sections
Primary tumor + vehicle
Primary tumor + DT
Cancer stem cells (stained for eGFP) Tumor cells (stained for RFP)
J
Gating cells
SSC-A (x1000)
FSC-A (x1000)
Cells 49.6%
Selecting single cells
Single cells_1 98.1%
FSC-H (x1000)
FSC-W (x1000)
Single cells_2 98.4%
SSC-H (x1000)
SSC-W (x1000)
Selecting the living cells
Living cells 98.9%
DAPI (450/50)
FSC-A (x1000)
Selecting RFP-Confetti+ cells
Cancer cells 99%
eGFP (525/50)
Confetti-RFP (610/20)
K
CRC Lgr5eGFP organoids
Lgr5+ (34.4%)
Lgr5- (39.1%)
eGFP (525/50)
FSC-A (x1000)
L
Primary tumor
Lgr5+ (6.1%)
Lgr5- (66.8%)
eGFP (525/50)
FSC-A (x1000)
M
****
Cancer cells (%)
67.5
6.7
Lgr5+
Lgr5-
N
CRC Control (DT)
Lgr5+ (0.2%)
Lgr5- (94.1%)
eGFP (530/30)
FSC-A (x1000)
O
Lgr5+
Lgr5-
Lgr5eGFP

Figure S1 related to Figure 1. (A) Representative example of a CRC organoid culture generated from VillinCre-ER^{T2}; *APC*$^{fl/fl}$; *KRAS*$^{LSL\text{-}G12D/+}$; *P53*$^{KO/KO}$; R26R-Confetti; Lgr5$^{DTR\text{-}eGFP}$ transgenic mice. **(B)** Representative example of a VillinCre-ER^{T2}; *APC*$^{fl/fl}$; *KRAS*$^{LSL\text{-}G12D/+}$; *P53*$^{KO/KO}$; R26R-Confetti; Lgr5$^{DTR\text{-}eGFP}$ endogenously expressing RFP-Confetti, in which CSCs are additionally labelled with membrane-bound *e*GFP (Lgr5$^{DTR\text{-}eGFP}$). **(C)** Experimental setup: VillinCre-ER^{T2}; *APC*$^{fl/fl}$; *KRAS*$^{LSL\text{-}G12D/+}$; *P53*$^{KO/KO}$; R26R-Confetti; Lgr5$^{DTR\text{-}eGFP}$ were orthotopically transplanted in recipient mice. Over time, organoids grow into primary intestinal tumors that spontaneously metastasize to liver and lungs. **(D)** Representative confocal images of orthotopic primary CRC. ROI = region of interest. **(E)** Representative confocal images of spontaneous liver (upper panel) and lung (lower panel) metastases derived from orthotopic CRC. **(F)** Representative confocal images of CRC Lgr5^{eGFP} organoids either untreated or treated with diphtheria toxin (DT) for 6 hours, stained with cleaved-Caspase-3 (red). Scales bars in A-F are all 100 µm. (**G)** Average number of cleaved Caspase-3 positive Lgr5^{+} or Lgr5^{-} cancer cells per CRC Lgr5^{eGFP} organoid, analyzed in different experimental conditions (untreated, DT-treated for either 3, 6 or 12 hours). **(H)** Diameter of CRC Lgr5^{eGFP} organoids included in the cleaved-Caspase-3 analysis. Red lines represent medians ± interquartile range. *P*-value calculated with one-way Anova test. **(I)** Representative examples of primary tumors either treated with vehicle (upper panel) or diphtheria toxin (DT) (lower panel). Dashed lines highlight tumor edges. Scale bars 500 µm (overview), 100 µm (zoom-in). (**J)** FACS strategy enabling the identification and sorting of Lgr5^{+} CSCs and Lgr5^{-} cancer cells in organoids and orthotopic primary tumors. **(K)** Representative example of FACS profile of CRC Lgr5^{eGFP} organoids. **(L)** Representative example of FACS profile of Lgr5^{+} CSCs and Lgr5^{-} cancer cells in primary orthotopic CRC. **(M)** Quantification of Lgr5^{+} CSCs and Lgr5^{-} cancer cells observed in orthotopic CRC by FACS (n=8). Data are presented as mean ± SEM, *P*-value ****< 0.0001, calculated with unpaired t-test with Welch's correction. **(N)** Representative example of FACS profile of CRC Lgr5^{eGFP} organoids subjected to selective ablation of Lgr5^{+} CSCs by diphtheria toxin treatment (DT), used to set the Lgr5^{-} gating. **(O)** Representative examples of sorted Lgr5^{+} CSCs and Lgr5^{-} cancer cells. Scales bars are all 100 µm.

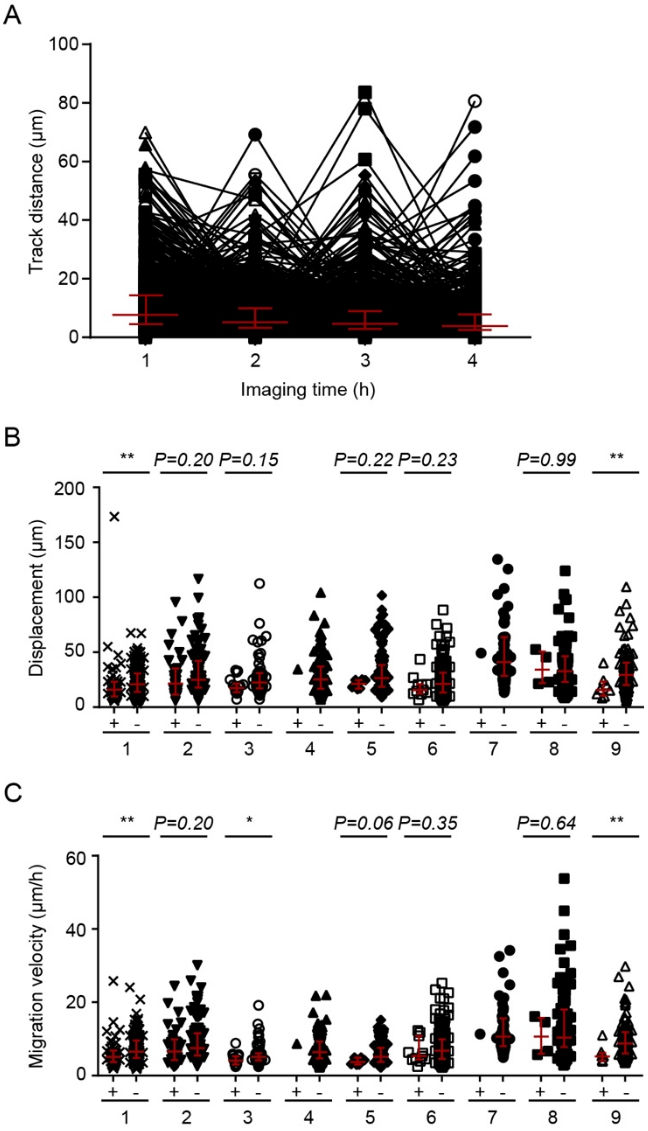

Figure S2 related to Figure 2. (A) Track distance of individual migratory cells followed by intravital imaging over the course of 4 hours. Each line represents an individual cell. Red lines indicate median (per time point) ± interquartile range. **(B-C)** *In vivo* displacement (B) and velocity (C) of escaping $Lgr5^{+}$ (+) CSCs and $Lgr5^{-}$ (-) cancer cells imaged in individual mice (n=9). Each data point represents a cell. *P*-value *< 0.05, **< 0.001. Red lines indicate median ± interquartile range. *P*-values calculated with Mann-Whitney U-test.

A

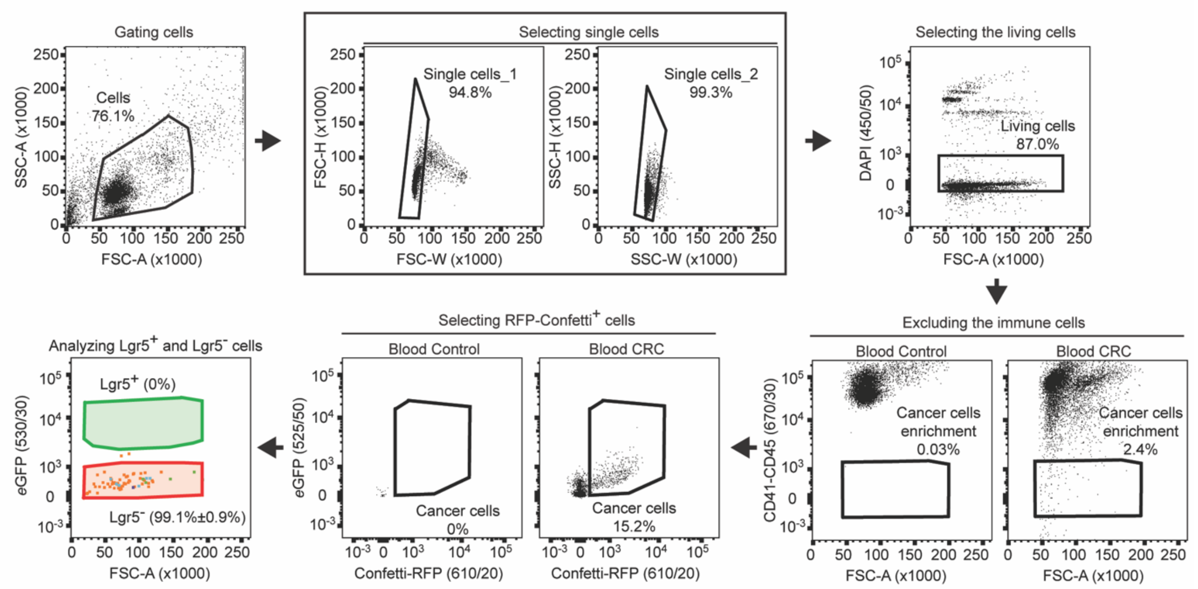

B

	Total count	Lgr5+	Lgr5-
Sample 1	5	0	5
Sample 2	1	0	1
Sample 3	6	0	6
Sample 4	56	0	54

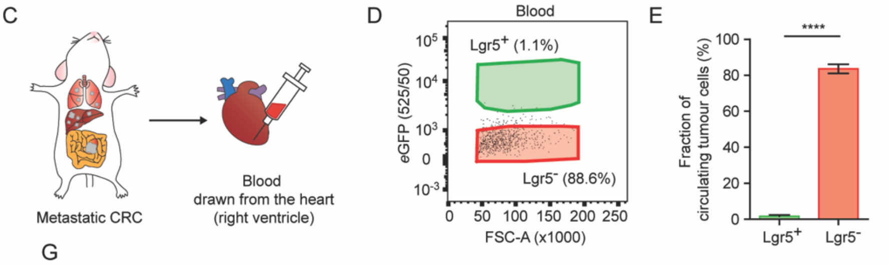

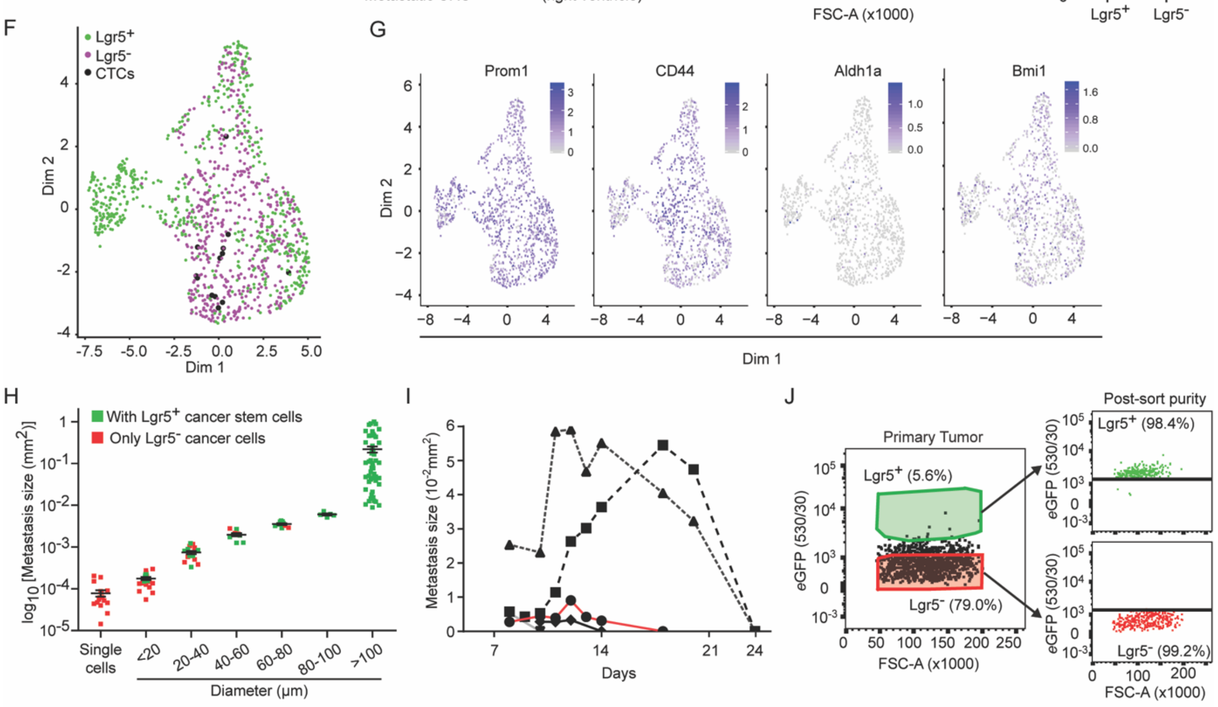

K

Replicate	Lgr5- post-sort purity (%)	Nr. of Lgr5+ cells injected as purity control	Nr. of metastasis in purity control	Nr. of metastasis in Lgr5+ sample	Nr. of metastasis in Lgr5- sample
1	100%	-	0	105	76
2	99.2%	48	0	52	17
3	100%	-	0	90	25
4	100%	-	0	77	24

Figure S3 related to Figure 3. (A) FACS strategy enabling distinctive sorting of circulating $Lgr5^+$ CSCs and $Lgr5^-$ cancer cells. Blood control = blood sample collected from a control NOD.Cg-$Prkdc^{scid}$ $Il2rg^{tm1Wjl}$/SzJ (NSG) mouse. **(B)** Table illustrating the total count of circulating $Lgr5^+$ CSCs and $Lgr5^-$ cancer cells in each blood sample obtained from the portal vein of mice bearing metastatic CRC tumors (see Figure 3A). The colour-coding links individual circulating tumor cells shown in Figure 3B to the corresponding blood sample. **(C)** Experimental setup: A metastatic intestinal tumor model was used to sample blood from the right ventricle of the heart. Blood was analyzed by FACS for the presence of circulating tumor cells. **(D)** Representative example of FACS profile of circulating $Lgr5^+$ CSCs and $Lgr5^-$ cancer cells detected in the systemic blood**. (E)** Quantification of circulating tumor cells found in the systemic blood (n=5). Data are presented as mean ± SEM, P-value ****< 0.0001, calculated with unpaired t-test with Welch's correction. **(F)** tSNE plot representing unbiased clustering of $Lgr5^+$ (green), $Lgr5^-$ (purple) in primary tumor, liver metastasis and circulating tumor cells (CTCs, black). **(G)** tSNE plots showing expression level of selected cancer stem cell marker genes in $Lgr5^+$, $Lgr5^-$ cancer cells of primary tumor, liver metastasis and circulating tumor cells clustered in (F). **(H)** Analysis of spontaneous liver metastases found in mice bearing orthotopic CRC tumors. Metastases are grouped per diameter range and plotted according to size (i.e. area represented in mm^2). Each individual point represents a metastatic lesion (n=132). The colour-coding identifies metastases composed of only $Lgr5^-$ cancer cells (red) and metastases containing $Lgr5^+$ CSCs (green). Black lines represent means ± SEM. **(I)** Diphtheria toxin (DT) treated metastasis plotted by their actual size (μm^2), followed over time by intravital imaging. The red line in represents the example shown in Fig. 3G. **(J-K)** Post-sort purity analysis on $Lgr5^+$ and $Lgr5^-$ sorted populations. (K) $Lgr5^-$ post-sort purity controls calculated in each experiment (n=4) that were performed for the analysis shown in Figure 3J to 3K. The table illustrates the equivalent number of $Lgr5^+$ cells injected in the purity control acceptor mouse and the number of metastases observed in purity control, $Lgr5^+$ and $Lgr5^-$ injected mice in each experimental replicate (n=4). Red highlights the example depicted in (J).

A

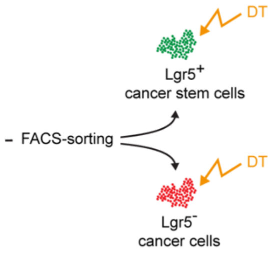

B

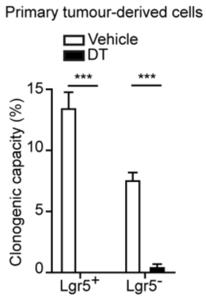

C

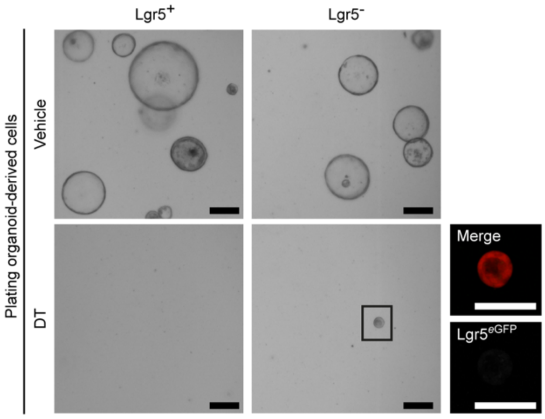

D

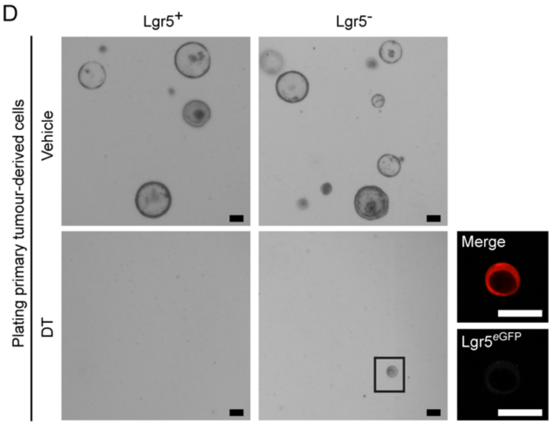

E

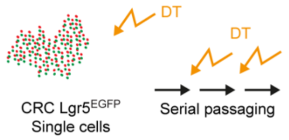

F

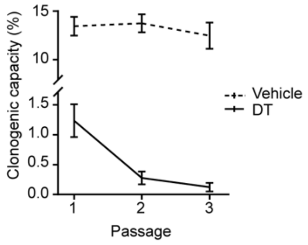

G

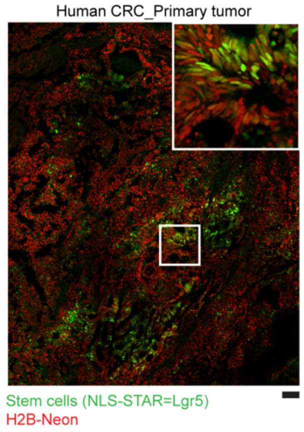

H

Human CRC_Dissemination

Nr. of animals with CTCs	Cell identity	Cell count
3/7 (43%)	STAR+	0
	STAR-	8

I

Human CRC_Metastasis ourgrowth

Founder cells	Nr. of animals with lesions	Nr. of liver metastasis
STAR+	0/5 (0%)	0
STAR-	3/5 (60%)	4

J

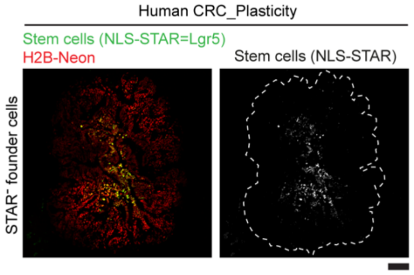

K

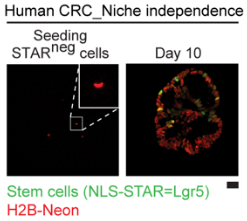

Figure S4 related to Figure 4. (A-B) Clonogenicity assay of sorted $Lgr5^+$ CSCs and $Lgr5^-$ cancer cells derived from colorectal orthotopic primary tumors. Single cells were subjected to either vehicle or diphtheria toxin (DT) treatment (n=3 independent experiments). Data were collected 6 days after plating. Values are presented as mean ± SEM, *P*-value ***< 0.0001, calculated with unpaired t-test with Welch's correction. **(C-D)** Representative example of organoid formation assay of sorted $Lgr5^+$ CSCs and $Lgr5^-$ cancer cells derived from either CRC organoid cultures (C) and orthotopic colorectal primary tumors 6 days after plating (D). Single cells were subjected to either vehicle or diphtheria toxin (DT) treatment. Scale bars, 100 µm. **(E-F)** Clonogenicity assay of CRC $Lgr5^{eGFP}$ organoid-derived cells. Single cells underwent serial passaging (every 6 days) while kept under constant vehicle or diphtheria toxin (DT) treatment (n=3 independent experiments). **(G)** Representative confocal image of a human orthotopic primary tumor (mutated in P53, PIK3CA, BRAF, ERBB3, RNF43). DsRed-expressing $Lgr5^+$ cells (using an ASCL2-responsive minigene (STAR) that labels $Lgr5^+$ CSCs with DsRed, Oost et al., 2018) are shown with a green lookup table for consistency with the images of our mouse model. All cancer cells express nuclear mNeon (red lookup table). **(H)** Total count of circulating $STAR^+$ CSCs and $STAR^-$ cancer cells in blood samples obtained from mice bearing human metastatic CRC tumors. **(I)** Number of metastatic foci derived from mesenteric vein injection of $STAR^+$ CSCs or $STAR^-$ cancer cells (n=5 biological replicates). **(J)** Representative confocal images of metastases generated from human $Lgr5^-$ cancer cells. **(K)** Representative example of human organoid formation assay of sorted $STAR^-$ cancer cells and cultured in minimum CRC medium (i.e. not containing stem cell-inducing growth factors). Scale bars, 100 µm.
