# Supplementary material for: Plasticity of Lgr5-Negative Cancer Cells Drives Metastasis in Colorectal Cancer
Source: Cell Stem Cell. 2020 Apr 2;26(4):569–578.e7. doi: 10.1016/j.stem.2020.02.008 (PMC7118369; doi:10.1016/j.stem.2020.02.008)
Supplement: Document S2. Article plus Supplemental Information [file mmc6.pdf]

Short Article

Cell Stem Cell

Plasticity of Lgr5-Negative Cancer Cells Drives Metastasis in Colorectal Cancer

Graphical Abstract

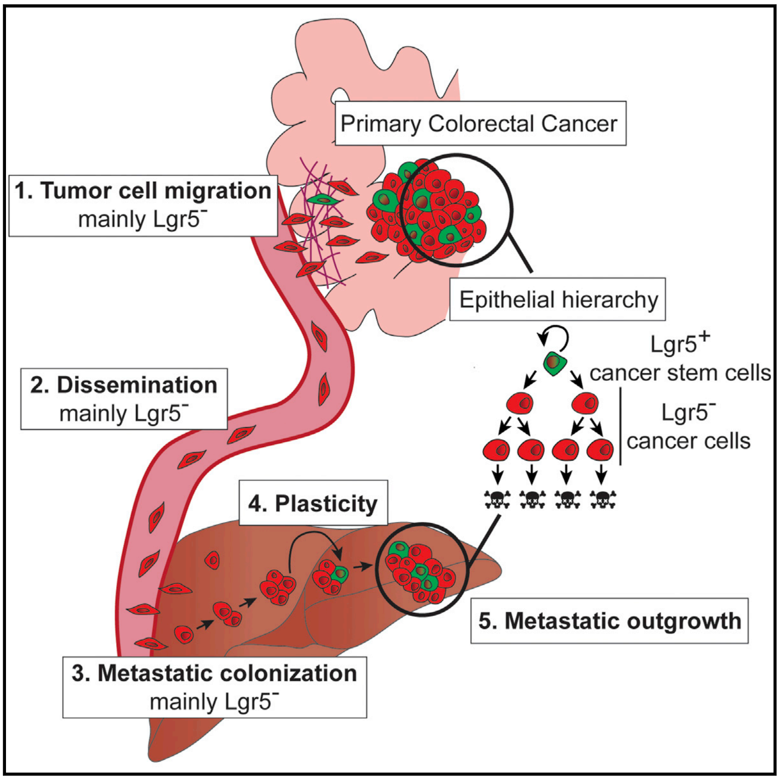

Authors

Arianna Fumagalli, Koen C. Oost, Lennart Kester, ..., Saskia J.E. Suijkerbuijk, Hugo J. Snippert, Jacco van Rheenen

Correspondence

j.v.rheenen@nki.nl

In Brief

Van Rheenen and colleagues study $Lgr5^+$ cancer stem cells during colorectal cancer metastasis. They demonstrate that the majority of metastases are seeded by $Lgr5^-$ cells, which upon arrival seed metastases in which $Lgr5^+$ cells appear. This plasticity can occur independently of stemness-inducing factors and is indispensable for long-term metastatic growth.

Highlights

- The majority of disseminating cells of colorectal cancer are $Lgr5^-$
- $Lgr5^-$ cancer cells are the main seeds of colorectal cancer metastatic lesions
- Long-term metastatic growth from $Lgr5^-$ cells requires appearance of $Lgr5^+$ cells
- $Lgr5^-$ metastases have the intrinsic capacity to re-establish the cellular hierarchy

Fumagalli et al., 2020, Cell Stem Cell 26, 569–578
April 2, 2020 © 2020 The Authors. Published by Elsevier Inc.
https://doi.org/10.1016/j.stem.2020.02.008

CellPress

Plasticity of Lgr5-Negative Cancer Cells Drives Metastasis in Colorectal Cancer

Arianna Fumagalli,[1,7,8] Koen C. Oost,[1,2,7] Lennart Kester,[1] Jessica Morgner,[1] Laura Bornes,[1] Lotte Bruens,[1,2] Lisa Spaargaren,[1] Maria Azkanaz,[1] Tim Schelfhorst,[1] Evelyne Beerling,[1] Maria C. Heinz,[2] Daniel Postrach,[1] Danielle Seinstra,[1] Anieta M. Sieuwerts,[4] John W.M. Martens,[4] Stefan van der Elst,[5] Martijn van Baalen,[3] Debajit Bhowmick,[3] Nienke Vrisekoop,[6] Saskia I.J. Ellenbroek,[1] Saskia J.E. Suijkerbuijk,[1] Hugo J. Snippert,[2] and Jacco van Rheenen[1,9,*]
[1]Department of Molecular Pathology, Oncode Institute, Netherlands Cancer Institute, 1066 CX Amsterdam, the Netherlands
[2]Molecular Cancer Research, Center for Molecular Medicine, Oncode Insitute, University Medical Center Utrecht, 3584 CX Utrecht, the Netherlands
[3]Flow Cytometry Facility, Netherlands Cancer Institute, 1066 CX Amsterdam, the Netherlands
[4]Department of Medical Oncology and Cancer Genomics Netherlands, Erasmus MC Cancer Institute, Erasmus University Medical Center, 3015 GD Rotterdam, the Netherlands
[5]Hubrecht Institute-KNAW & University Medical Center Utrecht, 3584 CT Utrecht, the Netherlands
[6]Department of Respiratory Medicine, Center of Translational Immunology, University Medical Center Utrecht, 3584 EA Utrecht, the Netherlands
[7]These authors contributed equally
[8]Present address: Oncode Institute, Princess Máxima Center for Pediatric Oncology, 3584 CS Utrecht, the Netherlands
[9]Lead Contact
*Correspondence: j.v.rheenen@nki.nl
https://doi.org/10.1016/j.stem.2020.02.008

SUMMARY

Colorectal cancer stem cells (CSCs) express Lgr5 and display extensive stem cell-like multipotency and self-renewal and are thought to seed metastatic disease. Here, we used a mouse model of colorectal cancer (CRC) and human tumor xenografts to investigate the cell of origin of metastases. We found that most disseminated CRC cells in circulation were Lgr5^{-} and formed distant metastases in which Lgr5^{+} CSCs appeared. This plasticity occurred independently of stemness-inducing microenvironmental factors and was indispensable for outgrowth, but not establishment, of metastases. Together, these findings show that most colorectal cancer metastases are seeded by Lgr5^{-} cells, which display intrinsic capacity to become CSCs in a niche-independent manner and can restore epithelial hierarchies in metastatic tumors.

INTRODUCTION

Epithelial tissues are hierarchically organized: a small pool of replicative immortal and self-renewing stem cells (SCs) give rise to a large population of more specialized cells that are replicative mortal (Beck and Blanpain, 2013). During development, SCs drive long-term growth, while during adult life they sustain tissues by driving turnover of short-lived specialized cells (Donati and Watt, 2015; Soteriou and Fuchs, 2018). This hierarchical organization is proposed to be maintained in tumors (Batlle and Clevers, 2017; Beck and Blanpain, 2013; Nassar and Blanpain, 2016). Indeed, cell-sorting and transplantation experiments showed that only a small pool of cancer cells, often referred to as cancer SCs (CSCs), have the ability to grow tumors in mice (Al-Hajj et al., 2003; Bonnet and Dick, 1997; Ricci-Vitiani et al., 2007). More recently, lineage-tracing experiments provided proof of the existence of CSCs in unperturbed tumors, including glioblastoma (Chen et al., 2012), squamous skin tumors (Driessens et al., 2012), colorectal cancer (CRC) (Cortina et al., 2017; Schepers et al., 2012; Shimokawa et al., 2017) and breast cancer (Zomer et al., 2013). In addition to the role in tumor growth and homeostasis, CSCs are thought to be important for metastasis. The essential role of CSCs in the metastatic process is reinforced by studies showing that metastasizing cells acquire SC properties, for example, through epithelial-to-mesenchymal transition (Guo et al., 2012; Mani et al., 2008; Ye et al., 2015). Indeed, selective depletion of CSCs in primary tumors protects from the appearance of distant metastases (de Sousa e Melo et al., 2017). The role of CSCs in metastasis would be particularly crucial if the tumor cell hierarchy is a rigid one-way route, from SCs to more specialized cells (Meacham and Morrison, 2013). However, epithelial hierarchical organization can be dynamic (Medema, 2013). For example, in healthy tissues, upon damage or selective ablation of the SC population, more specialized cells can acquire SC traits (Ritsma et al., 2014; Tetteh et al., 2016; Tian et al., 2011; van Es et al., 2012). This process of cellular plasticity ensures repopulation of impaired SC niches and reestablishment of tissue homeostasis. Whether cellular plasticity also plays an important role in metastasis is unknown. In this study, we dissect the different steps of the metastatic cascade (cell migration, intravasation, metastatic seeding, and outgrowth) and uncover the roles of CSCs, non-CSCs, and cellular plasticity in metastasis.

This is an open access article under the CC BY license (http://creativecommons.org/licenses/by/4.0/).

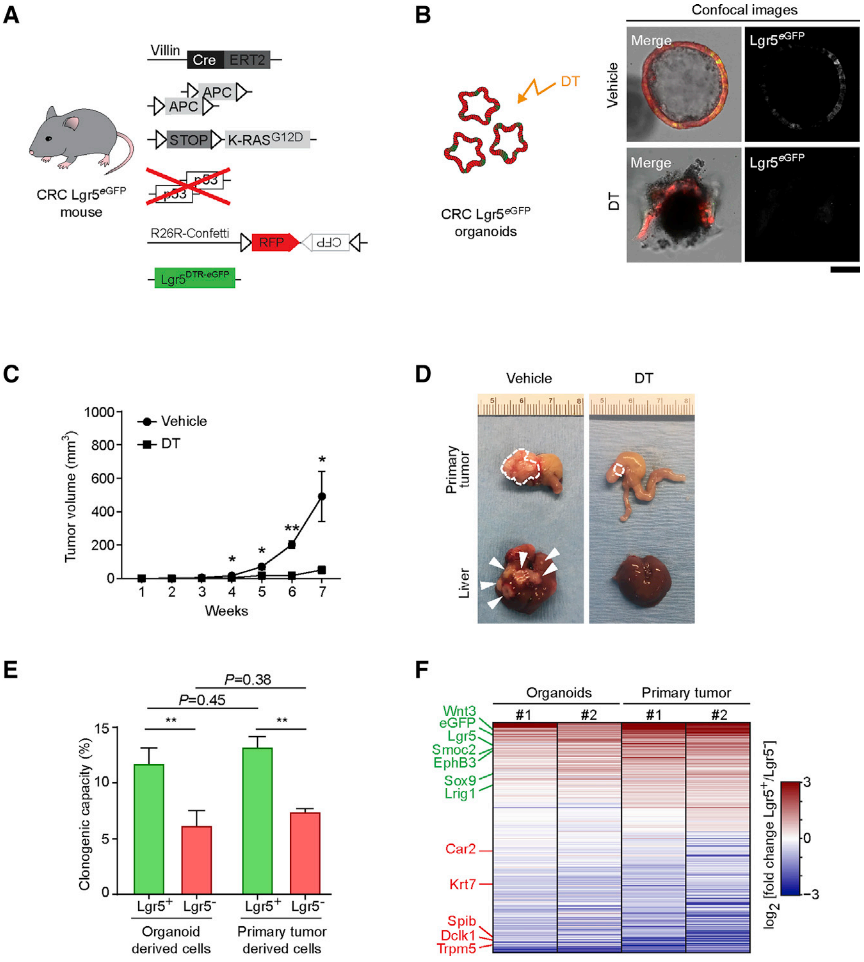

Figure 1. Generation of a Colorectal Cancer Mouse Model to Visualize Lgr5$^+$ CSCs

(A) Schematic overview of the inducible fluorescent CRC mouse model generated to visualize Lgr5$^+$ CSCs.

(B) Diphtheria toxin (DT) treatment of CRC Lgr5^{eGFP} organoids. Representative confocal images of the effect of vehicle or DT on organoid growth. Scale bar, 100 μm.

(C) Prolonged diphtheria toxin (DT) treatment of mice orthotopically transplanted with CRC Lgr5^{eGFP} organoids. Average tumor growth upon vehicle or DT treatment (n = 5). *p < 0.05 and **p < 0.001. Data are presented as mean ± SEM; p values were calculated using the Mann-Whitney U test.

(D) Representative examples of primary tumors and livers of mice subjected to either vehicle or DT treatment for 8 weeks. Dashed lines highlight primary tumor edges, arrowheads indicate macroscopic metastatic lesions.

(E) Clonogenicity assay of sorted Lgr5$^+$ CSCs and Lgr5$^-$ cancer cells derived from CRC organoid cultures and orthotopic colorectal primary tumors. Data were collected 6 days after plating (n = 3 independent experiments). Values are presented as mean ± SEM; p values calculated using the unpaired t test with Welch's correction.

(F) Heatmap of differentially expressed transcripts in cancer cells derived from two independent biological replicates of organoids and orthotopic primary tumors. Genes marked in green are known to be upregulated in intestinal stem cells; genes marked in red are known intestinal differentiated cell markers.

RESULTS

Lgr5^{eGFP} Labels Functional Stem Cells in Colorectal Tumors

CRC models provide a unique opportunity to clarify the role of CSCs and non-CSCs in the metastatic process, because hierarchical organization is maintained during disease progression and functional CSCs are marked by Lgr5 expression (Cortina et al., 2017; de Sousa e Melo et al., 2017; Schepers et al., 2012; Shimokawa et al., 2017). To study the role of CSCs in metastasis, we combined the VillinCre-ER^{T2}; APC$^{fl/fl}$; KRAS$^{LSL-G12D}$; TP53$^{KO/KO}$ colorectal tumor mouse model with Lgr5$^{DTR/eGFP}$ (de Sousa e Melo et al., 2017) and Confetti (Snippert et al., 2010) fluorescent mouse models (Figure 1A). Upon injection of tamoxifen, the entire intestinal tract of these mice becomes tumorigenic, and these animals reach the humane endpoint before metastases are formed. Just before the humane endpoint, we isolated the tumorigenic colons of these mice and established primary CRC organoids that consist of cancer cells expressing RFP-Confetti and of CSCs that additionally express Lgr5-driven eGFP and diphtheria toxin receptor (DTR) (referred as CRC Lgr5^{eGFP} organoids; Figures S1A and S1B). As previously described, orthotopic transplantation of these murine CRC organoids led to the formation of a single colorectal primary tumor that spontaneously metastasizes to liver and lungs (Figures S1C–S1E) (Fumagalli et al., 2017, 2018; Roper et al., 2017; Tauriello et al., 2018). In line with previous findings (Cortina et al., 2017; de Sousa e Melo et al., 2017; Shimokawa et al., 2017), we found that Lgr5 is a good marker of the functional SCs both in organoids and tumors, because selective ablation of the Lgr5$^+$ CSCs by diphtheria toxin (DT) (Figures S1F–S1I) caused organoids to collapse and prevented tumor growth and metastasis (Figures 1B–1D). Next, we developed a flow cytometry strategy to isolate Lgr5$^+$ CSCs and Lgr5$^-$ cancer cells from both organoids and primary tumors (Figures S1J–S1M). Analysis of DT-treated CRC Lgr5^{eGFP} organoids, in which Lgr5$^+$ CSCs are selectively depleted, and post-sort confocal microscopy on untreated CRC Lgr5^{eGFP} organoids confirmed that our gating strategy allowed to reliably isolate Lgr5$^+$ and Lgr5$^-$ cancer cells (Figures S1N and S1O). Clonogenic assays confirmed isolation of functional Lgr5$^+$ CSCs, as these cells were twice as clonogenic as Lgr5$^-$ cancer cells (Figure 1E). Moreover, isolated Lgr5$^+$ CSCs showed higher expression of known intestinal SC markers and lower expression of differentiated markers than the Lgr5$^-$ cancer cells (Figure 1F) (de Sousa e Melo et al., 2017; Shimokawa et al., 2017). Of note, we found a very significant overlap in significantly differentially expressed genes between Lgr5$^+$ and Lgr5$^-$ cells from organoids and orthotopic

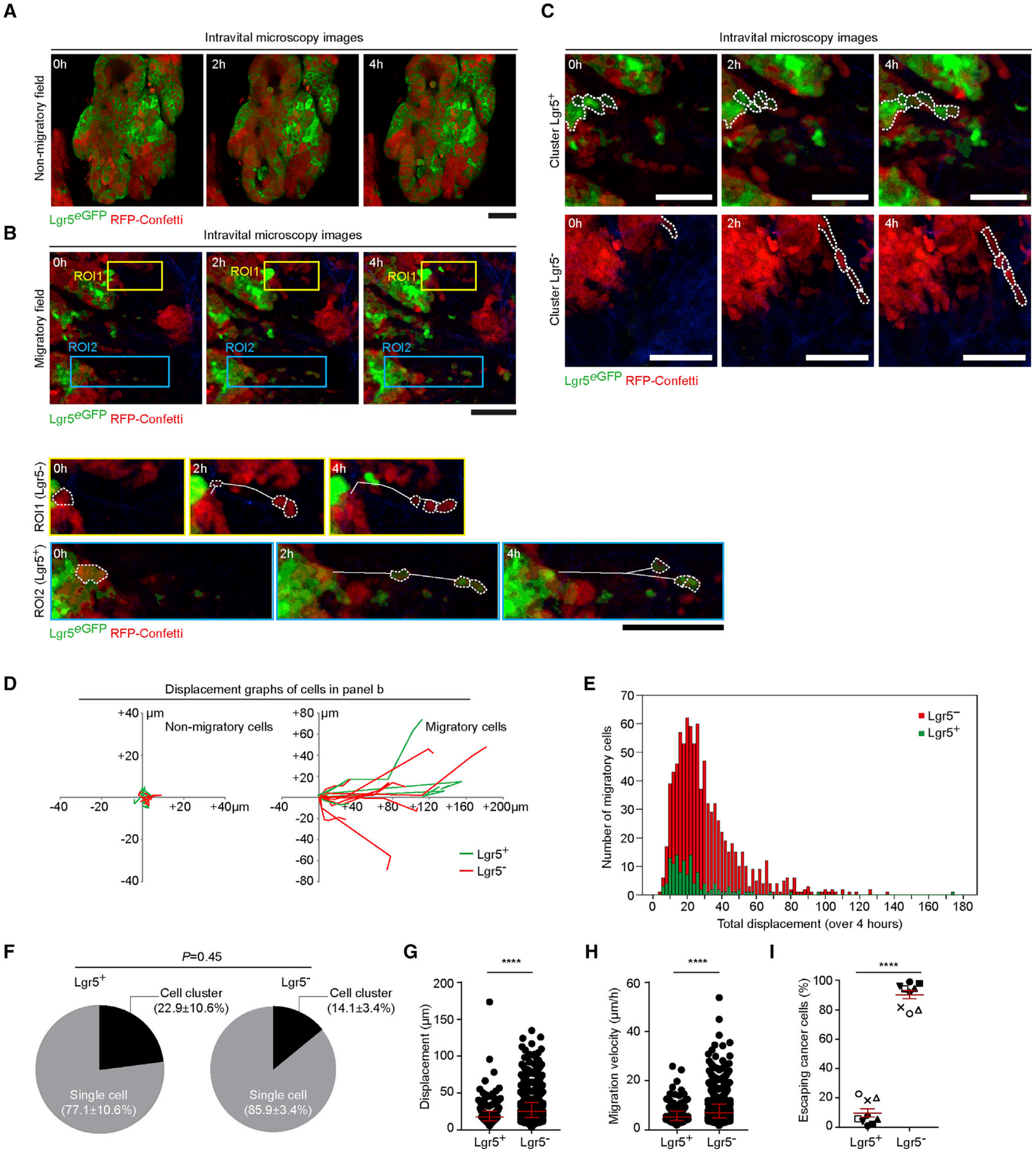

Figure 2. The Majority of Cells Escaping the Primary Colorectal Tumors Are Lgr5⁻

(A) Representative time-lapse intravital images of a non-migratory field within a CRC primary tumor. Note that the resolution of *in vivo* images is lower than of *ex vivo* images because of great depth of imaging in living animals. Scale bar, 50 μm.

(B) Time-lapse intravital images of a migratory field showing primary tumor cell migration of $Lgr5^-$ and cancer cells (ROI, region of interest 1) and $Lgr5^+$ CSCs (ROI2). Dashed lines highlight the migratory cells, continuous lines mark the migratory tracks overtime. Scale bars, 50 μm. See also Videos S1 and S2.

(C) Representative time-lapse intravital images of $Lgr5^+$ CSCs (upper panel) and $Lgr5^-$ cancer cells (lower panel) moving as cell clusters (see also Videos S3 and S4). Scale bars, 50 μm.

(D) Display of the migratory tracks of $Lgr5^+$ CSCs and $Lgr5^-$ cancer cells observed in (A) (left) and in (B) (right).

(E) Distribution of total displacement of $Lgr5^+$ (green) and $Lgr5^-$ (red) migratory cells over a period of 4 h.

(legend continued on next page)

primary tumors (hypergeometric test p value < 10^{-100}), indicating that $Lgr5^+$ and $Lgr5^-$ cancer cells maintain their specific cellular identity when analyzed either *in vitro* or *in vivo*.

The Majority of Migratory Tumor Cells Are Lgr5 Negative

Using the developed orthotopic transplantation mouse model, we addressed whether $Lgr5^+$ CSCs and $Lgr5^-$ cancer cells have differential metastatic behavior. In order to metastasize, cancer cells first need to acquire traits that enable them to leave the primary tumor (Hanahan and Weinberg, 2011). To test which cancer cells escape from the primary site, we filmed the behavior of $Lgr5^+$ CSCs and $Lgr5^-$ cancer cells *in vivo* using multiphoton microscopy (Figure 2; Videos S1, S2, S3, and S4). Consistent with other tumor models (Beerling et al., 2016; Hirata et al., 2015; Patsialou et al., 2013), in some tumor lobes no migration was observed, whereas in other lobes individual and clusters of $Lgr5^+$ CSCs and $Lgr5^-$ cancer cells invaded into the non-labeled stroma (n = 9 mice, 73 movies, 1,064 migratory events; Figures 2A–2C; Figure S2A; Videos S1, S2, S3, and S4). Analysis of the migratory cells (i.e., cells that displaced more than half a cell diameter in 4 h; Figures 2D and 2E) revealed that $Lgr5^+$ CSCs and $Lgr5^-$ cancer cells mainly escaped as single cells and less frequently as cell clusters (Figure 2F). Moreover, $Lgr5^-$ cancer cells might have a slightly higher displacement and velocity than $Lgr5^+$ CSCs (Figures 2G and 2H; Figures S2B and S2C). Surprisingly, of the 1,064 migratory cells that escaped from the primary tumors, the majority were $Lgr5^-$ (Figures 2E and 2I; 90.3% ± 2.8%, $p < 0.0001$), suggesting that most of the escaping cells are in a more differentiated state.

The Vast Majority of Disseminating Cells in the Circulation Are Lgr5 Negative

To determine whether both $Lgr5^+$ CSCs and $Lgr5^-$ cancer cells were able to enter the blood circulation, we drew blood from the portal vein of mice bearing metastatic CRC to analyze CTCs directly draining from the primary tumor using flow cytometry (Figure 3A; Figure S3A). In all animals (n = 4) we detected only $Lgr5^-$ circulating cancer cells (Figure 3B; Figure S3B). We extended the analysis by collecting systemic oxygen-poor blood from the right ventricle of the heart of a second batch of metastatic CRC mice to analyze CTCs coming from metastases and observed that also in this case the vast majority of circulating cancer cells were $Lgr5^-$ (98.4% ± 0.6%, n = 5; Figures S3C–S3E). Importantly, single-cell mRNA sequencing validated that the CTCs displayed an expression profile closely related to $Lgr5^-$ cancer cells and did not show enriched expression of other CSC marker genes, such as Prom1 (i.e., CD133), CD44, Aldh1a, and Bmi1 (Figure 3C; Figures S3F and S3G). Combined these data suggest that the disseminating population of cancer cells is dominated by $Lgr5^-$ cancer cells. Indeed, when we analyzed the livers of these mice, all single-cell metastases were $Lgr5^-$ (n = 6 mice; Figures 3D and 3E; Figure S3H).

Stem Cell Plasticity Is Required for Metastatic Seeding in the Liver

To understand whether escaping $Lgr5^-$ cancer cells could initiate metastatic lesions, we analyzed larger metastases in the livers. Although all single-cell and the majority of smaller metastases were devoid of $Lgr5^+$ CSCs, all larger metastases (lesions larger than 80 μm in diameter) contained a population of $Lgr5^+$ CSCs (Figures 3D and 3E; Figure S3H). To test whether $Lgr5^-$ cancer cells could give rise to full-blown metastatic lesions, we monitored the real-time outgrowth of $Lgr5^-$ cancer cells seeding in the liver, by tracking the same metastatic lesions with repeated multi-day intravital imaging through an abdominal imaging window (Figure 3F). In all small lesions initially containing only $Lgr5^-$ cancer cells, we observed that some of the $Lgr5^-$ tumor cells underwent plasticity and acquired $Lgr5^{eGFP}$ expression over time (Figure 3F). Next, we neutralized this plasticity by ablating newly formed $Lgr5^+$ CSCs in early metastatic lesions and observed by intravital imaging that metastasis composed by only $Lgr5^-$ cancer cells stopped growing over time and ultimately regressed (Figures 3G and 3H; Figure S3I). Indeed, after 4 weeks of DT treatment, no metastases could be observed (Figures 3I and 3J). Combined this shows that the appearance of $Lgr5^+$ CSCs is indispensable for the outgrowth of metastases founded by $Lgr5^-$ cancer cells.

To compare the efficiency of $Lgr5^-$ cancer cells and $Lgr5^+$ CSCs to initiate metastases, we injected equal amounts of sorted $Lgr5^+$ CSCs and $Lgr5^-$ cancer cells into the mesenteric vein, which are transported through the portal vein to the liver. Four weeks later we found that, in addition to $Lgr5^+$ CSCs, $Lgr5^-$ cancer cells were able to form metastases that displayed similar morphology, though with slightly lower efficiency (Figures 3K–3O; Figures S3J and S3K; n = 4 mice). Given that most disseminating cells were $Lgr5^-$ and had the ability to initiate metastatic growth, our data reveal that the majority of metastases are seeded by $Lgr5^-$ cancer cells.

$Lgr5^-$ Clones Have the Intrinsic Capacity to Restore the Cellular Hierarchy

Because growth of metastases seeded by $Lgr5^-$ cancer cells requires cells to become $Lgr5^+$, we next addressed what is driving this plasticity. It has been suggested that cancer cells can acquire SC properties when they are surrounded by a SC niche, such as fibroblasts and immune cells, that provides stemness-inducing signals (Sato and Clevers, 2013). To test whether SC-inducing microenvironmental factors are deterministic for cancer cell plasticity, we set up an organoid-forming assay. In these pure epithelial culture systems, the three-dimensional (3D) microenvironment was mimicked by

(F) Characterization of $Lgr5^+$ CSCs and $Lgr5^-$ cancer cells migratory mode (i.e., cells escaping as single cells or as cell clusters (i.e., maintaining cell-cell contact). Data are presented as mean ± SEM (n = 9); p values were calculated using the unpaired t test with Welch's correction.

(G and H) *In vivo* displacement (G) and velocity (H) of escaping $Lgr5^+$ CSCs and $Lgr5^-$ cancer cells. Each data point represents a cell. ****$p < 0.0001$. Red lines indicate median ± interquartile range; p values were calculated using the Mann-Whitney U test.

(I) Fraction of escaping $Lgr5^+$ CSCs and $Lgr5^-$ cancer cells observed *in vivo* in individual mice. Different shapes represent different animals (n = 9). Each data point indicates the average value per animal. ****$p < 0.0001$. Red lines indicate mean ± SEM; p value was calculated using the unpaired t test.

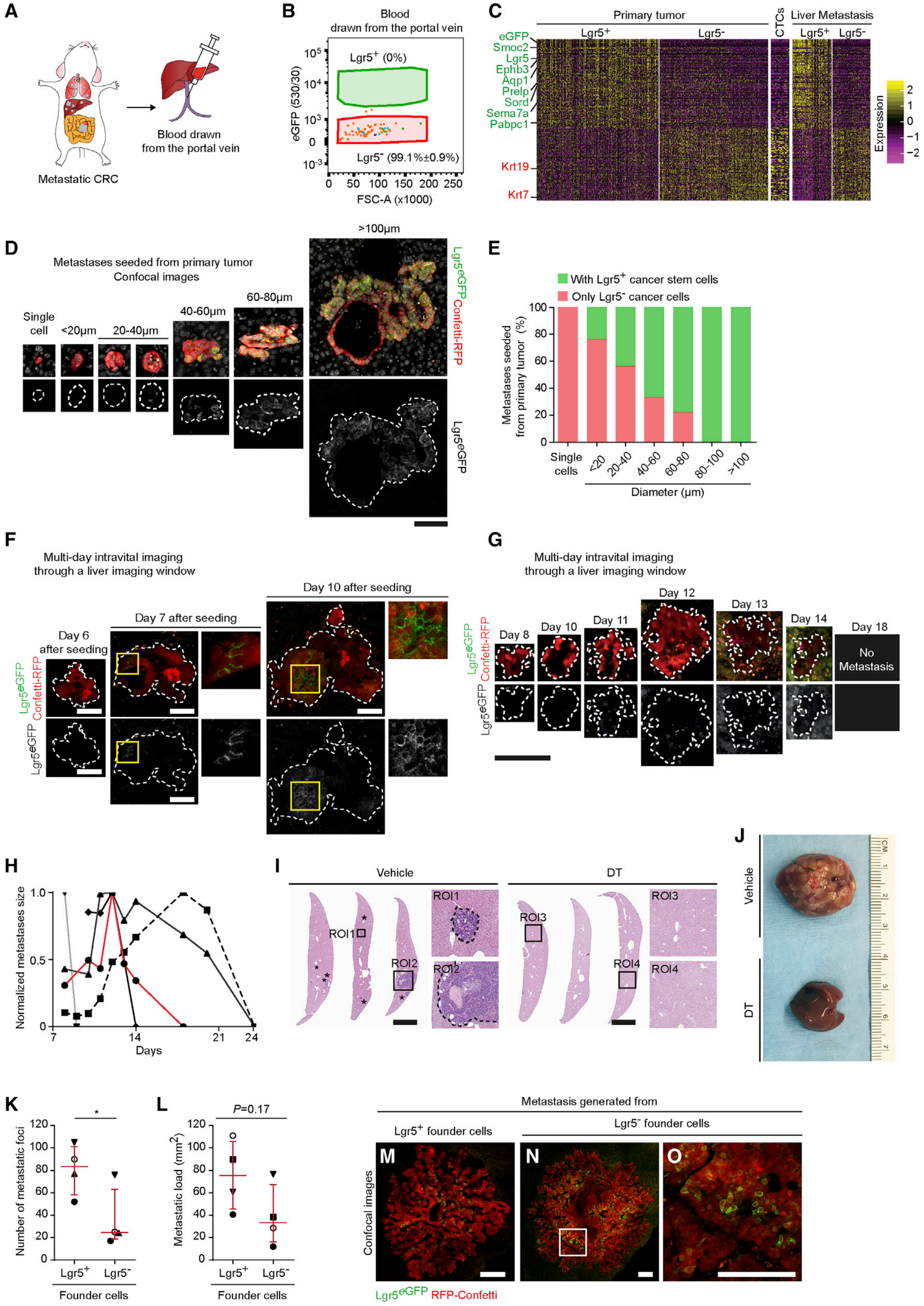

(legend on next page)

Basement Membrane Extract (BME) and medium providing all the essential SC-inducing factors present in the intestinal SC niche, such as Wnt, R-spondin, and EGF (Sato and Clevers, 2013). By simply removing one or more of these niche factors, it is possible to test whether intestinal epithelial cells and their dynamics are dependent on essential microenvironmental signals (Drost et al., 2015; Fujii et al., 2016). We isolated single $Lgr5^{-}$ circulating cancer cells from the blood and tested whether they could be cultured in medium without any SC-inducing factors. Interestingly, in this minimal medium we were able to grow organoids from $Lgr5^{-}$ circulating cancer cells, and also here we observed the appearance of $Lgr5^{+}$ CSCs over time (Figures 4A and 4B). Moreover, these $Lgr5^{+}$ cancer cells were functional SCs, as selective ablation of these cells by acute or chronic DT treatment in both organoid-derived and primary tumor-derived cells abolished organoid outgrowth (Figures 4C; Figures S4A–S4F). To exclude that residual growth factors in the BME matrix could trigger cancer cell plasticity in our culture system, we confirmed these data in an engineered 3D matrix consisting of growth factor-free synthetic hydrogel (Figures 4D and 4E). Finally, to test whether plasticity can be enhanced by microenvironmental factors that are released by cancer associated fibroblasts, we cultured $Lgr5^{-}$ cells in minimal medium supplemented with HGF, FGF, IL4, and IL13 (Chen et al., 2018; Knuchel et al., 2015; Lenos et al., 2018; Todaro et al., 2007, 2014). Indeed, we observed enhanced plasticity (i.e., appearance of $Lgr5^{+}$ cells) in medium supplemented with HGF and FGF compared with a basal level of plasticity in minimal CRC medium (Figure 4F). These data suggest that clones seeded by $Lgr5^{-}$ cancer cells have the intrinsic capacity to re-establish the cellular hierarchy, even in conditions devoid of microenvironmental SC-inducing signals, and that this plasticity can be further enhanced by microenvironmental factors such as HGF and FGF.

DISCUSSION

Although epithelial hierarchy is often seen as a static, one-directional route from SCs to differentiated cells, our study draws a more dynamic picture of CRC, particularly of the metastatic setting. We showed that, in addition to CSCs, non-CSCs are also capable of giving rise to colorectal liver metastases. Although CSCs are present in the primary tumor and in the migratory population (Figure 2), we did not detect them in circulation. As already shown for healthy intestine (Sato et al., 2011; Serra et al., 2019), CSCs may lose Lgr5 expression and stemness once they are not supported by the surrounding SC niche and therefore may be forced into a $Lgr5^{-}$ state. Regardless of the origin of the disseminating non-CSCs, non-CSCs are in fact the major seeding cells, while conversion to CSCs at the metastatic site is required for efficient metastatic outgrowth. Therefore, cancer cell plasticity, rather than seeding of CSCs, is a key step in the formation of CRC metastases. To confirm the occurrence of plasticity in human tissues, we orthotopically transplanted human tumor organoids characterized by SC Ascl2 reporter that labels $Lgr5^{+}$ CSCs (i.e., STAR probe; see Oost et al., 2018) (Figure S4G). Similar to their murine counterpart, these experiments showed that the major disseminating population (CTCs) was $Lgr5^{-}$ (Figure S4H). Moreover, human $Lgr5^{-}$ cells efficiently seed metastases that contained $Lgr5^{+}$ CSCs (Figures S4I and S4J). Plasticity in human cancer cells can happen independently of SC-inducing factors, as $Lgr5^{+}$ CSCs appeared in organoids that were seeded by $Lgr5^{-}$ cells and cultured in minimal CRC medium (Figure S4K). These experiments showed that differentiated cancer cells are able to form metastases, are plastic, and can re-establish the cellular hierarchy also in the human setting (Figures S4H–S4K). Cellular plasticity of epithelial cells has already been reported in healthy intestine (Ritsma et al., 2014; Tetteh et al., 2016;

Figure 3. $Lgr5^{-}$ Cancer Cells Are the Disseminating Cells in CRC

(A) Experimental setup: mice bearing metastatic CRC were used to sample blood from the portal vein. Blood was analyzed using FACS for the presence of circulating tumor cells.

(B) Cumulative FACS profile of circulating $Lgr5^{+}$ CSCs and $Lgr5^{-}$ cancer cells (n = 4). The color coding links individual circulating tumor cells to the corresponding blood sample in Figure S3B.

(C) Heatmap of differentially expressed genes in $Lgr5^{+}$ and $Lgr5^{-}$ cancer cells isolated from primary tumors, CTCs, and liver metastasis. Genes marked with green are known to be upregulated in intestinal stem cells; genes marked in red indicate known intestinal differentiated cell markers.

(D) Representative confocal images of spontaneous liver metastases grouped per diameter range. Dashed lines highlight the metastasis edges. Scale bar, 100 μm.

(E) Metastases are subdivided in lesions composed of only $Lgr5^{-}$ cancer cells (red) or lesions containing $Lgr5^{+}$ CSCs (green) and grouped per diameter range (the analysis includes 132 metastatic lesions, n = 6).

(F) Intravital multi-day imaging of liver metastases hatching from $Lgr5^{-}$ cancer cells. Dashed lines highlight metastasis edges. Yellow boxed areas are enlarged next to the corresponding panels. Scale bars, 50 μm.

(G) Representative images of a liver metastasis followed overtime by intravital imaging demonstrating growth and regression upon depletion of $Lgr5^{+}$ CSCs via diphtheria toxin (DT) administration. Dashed lines highlight metastasis border. Scale bar, 100 μm.

(H) Size of DT-treated metastatic lesions followed by intravital imaging (n = 5), normalized to the first time point. The red line represents the example shown in (G).

(I) Representative histochemistry images of livers of mice subjected mesenteric vein injection of FACS-sorted $Lgr5^{-}$ cancer cells and treated with either vehicle or DT treatment for 4 weeks. ROI, region of interest. Asterisks indicate metastatic foci; dashed lines highlight metastasis edges. Boxed areas are enlarged next to the corresponding overview images. Scale bars, 500 μm.

(J) Representative examples of livers of mice of mice subjected mesenteric vein injection of CRC $Lgr5^{eGFP}$ and treated with either vehicle or DT treatment for 4 weeks.

(K) Number of metastatic foci derived from mesenteric vein injection of $Lgr5^{+}$ CSCs or $Lgr5^{-}$ cancer cells.

(L) Metastatic load upon injection of $Lgr5^{+}$ CSCs or $Lgr5^{-}$ cancer cells. Similar shapes represent paired experiments (n = 4). Data are presented as median with interquartile range; p values were calculated using the paired t test ($^{*}p < 0.05$).

(M–O) Representative examples of metastases generated from $Lgr5^{+}$ CSCs (M) and $Lgr5^{-}$ cancer cells (N), boxed area enlarged in (O). Scale bars, 100 μm.

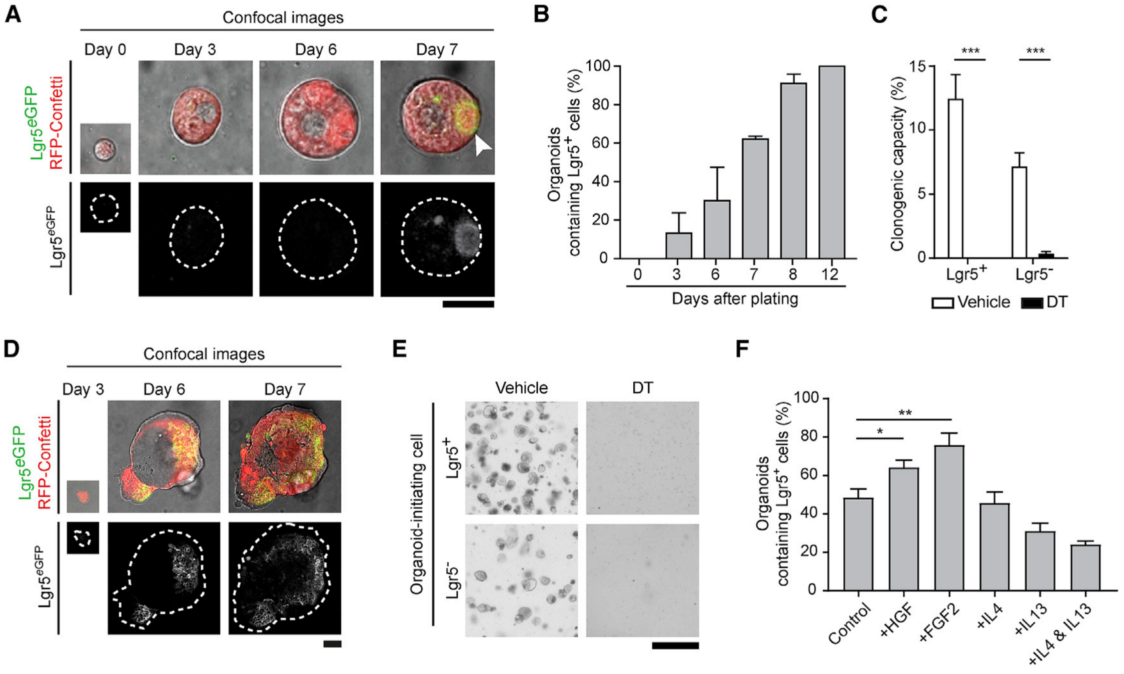

Figure 4. Disseminating $Lgr5^-$ Cancer Cells Undergo Niche-Independent Cellular Plasticity

(A) Representative example of organoid formation assay of sorted $Lgr5^-$ circulating cancer cells. Dashed lines highlight organoids edges. Arrowhead indicates the appearance of a $Lgr5^+$ CSC. Scale bar, 20 μm.

(B) Quantification of cancer cell plasticity (i.e., emergence of $Lgr5^+$ CSCs) in the organoid-forming assay of sorted $Lgr5^-$ circulating cancer cells grown in minimal CRC medium. Data are presented as mean ± SEM (n = 3 independent experiments).

(C) Clonogenicity assay in minimal CRC medium of sorted $Lgr5^+$ CSCs and $Lgr5^-$ cancer cells derived from CRC organoid cultures. Single cells were subjected to either vehicle or diphtheria toxin (DT) treatment (n = 3 independent experiments). Data were collected 6 days after plating. Values are presented as mean ± SEM; ***$p < 0.0001$, calculated using the unpaired t test with Welch's correction.

(D) Representative example of organoid formation assay of sorted $Lgr5^-$ cancer cells cultured in synthetic hydrogel. Dashed lines highlight organoids edges. Scale bar, 50 μm.

(E) Representative images of the effect of vehicle or diphtheria toxin (DT) on sorted $Lgr5^+$ CSCs and $Lgr5^-$ cancer cells cultured in synthetic hydrogel 6 days after plating (n = 3 independent experiments). Scale bar, 200 μm.

(F) Quantification of cancer cell plasticity (i.e., emergence of $Lgr5^+$ CSCs) of sorted $Lgr5^-$ cancer cells grown in either minimal CRC medium (control) or medium containing FGF-2 (10 ng/mL), HGF (75 ng/mL), IL4 (20 ng/mL), IL13 (100 ng/mL), and IL4 and IL13. n = 3 independent experiments. *$p < 0.05$ and **$p < 0.001$, calculated using the unpaired t test. Data are represented as mean ± SEM.

Tian et al., 2011; van Es et al., 2012) and as recently reviewed by de Sousa e Melo and de Sauvage (2019). Upon targeted depletion of the healthy $Lgr5^+$ SC population, more specialized $Lgr5^-$ cells enter the SC niche and are instructed to revert to $Lgr5^+$ SCs, thereby restoring tissue homeostasis (Ritsma et al., 2014; Tetteh et al., 2016; Tian et al., 2011; van Es et al., 2012). The intestinal SC niche consists of supporting cells producing stemness-inducing factors, such as Wnt, R-spondin, and EGF. Similar plasticity processes have been observed in primary CRC tumors, and various microenvironmental signals have been highlighted as responsible for the switch of non-CSCs to CSCs (Lenos et al., 2018; Schwitalla et al., 2013; Vermeulen et al., 2010). However, our data also suggest that cancer cell plasticity can be triggered independently of stemness-inducing factors provided by SC niches. Nevertheless, we also show that factors released by fibroblasts, such as FGF and HGF, can enhance plasticity. Regardless of this enhancement, our data suggest that specifically targeting CSCs and/or the SC-inducing niche, as previously proposed (de Sousa e Melo and de Sauvage, 2019), may not be enough to completely prevent metastatic disease, which is the leading cause of cancer-related death. Instead, endogenous cellular plasticity should be co-targeted to inactivate any potential seeds of metastasis, and future studies should aim to uncover microenvironment-independent mechanisms leading to cell plasticity.

STAR★METHODS

Detailed methods are provided in the online version of this paper and include the following:

- KEY RESOURCES TABLE
- LEAD CONTACT AND MATERIALS AVAILABILITY
- EXPERIMENTAL MODEL AND SUBJECT DETAILS
  - Generation of a colorectal cancer genetic mouse model
  - Isolation and culturing of mouse organoids
  - Acceptor mice for transplantation experiments
  - Human organoids

- METHOD DETAILS
  - Orthotopic transplantation of murine organoids
  - Orthotopic transplantation of human organoids
  - Flow cytometry on organoids
  - Flow cytometry on primary mouse material
  - Flow cytometry on human xenografts
  - Immunohistochemistry and fluorescent imaging
  - *In vivo* time-lapse imaging of primary tumors
  - Multi-day imaging of liver metastasis
  - Mesenteric vein injection
  - Intrahepatic injection
  - Organoid formation assays
  - *In vitro* Diphtheria Toxin treatment
  - *In vivo* Diphtheria Toxin treatment
  - Hydrogel assay
  - Comparison of $Lgr5^+$ and $Lgr5^-$ metastatic efficiency
  - Comparison of $STAR^+$ and $STAR^-$ metastatic efficiency
- QUANTIFICATION AND STATISTICAL ANALYSIS
  - Flow cytometry
  - Fluorescent images (fixed samples)
  - Intravital imaging of primary tumors
  - Intravital imaging of liver metastasis
  - Organoid formation assays
  - Metastasis scoring
  - Single-cell mRNA sequencing
- DATA AND CODE AVAILABILITY

SUPPLEMENTAL INFORMATION

Supplemental Information can be found online at https://doi.org/10.1016/j.stem.2020.02.008.

ACKNOWLEDGMENTS

We thank Anko de Graaff and the Hubrecht Imaging Centre and Amalie Dick for technical support with imaging. We thank members of the van Rheenen group for critically reading the manuscript, Life Science Editors for editing this manuscript, and Frederic J. de Sauvage for sharing the Lgr5-DTR-GFP mouse. This work was financially supported by Dutch Cancer Society Fellowship BUIT-2013-5847 (to S.J.E.S.), European Molecular Biology Organization (EMBO) fellowship ALTF 202-2016 (to J.M.), the Netherlands Organization of Scientific Research (NWO) (Veni grant 863.15.011 to S.I.J.E.), CancerGenomics.nl (to J.v.R., A.M.S., and J.W.M.M.), European Research Council Grant CANCER-RECURRENCE 648804 (to J.v.R.), the Doctor Josef Steiner Foundation (to J.v.R.), and the European Union's Horizon 2020 research and innovation program under Marie Skłodowska-Curie grant agreement 642866 (both to J.v.R.).

AUTHOR CONTRIBUTIONS

A.F. performed the experiments. K.C.O. performed part of the revision experiments with the help of M.A., E.B., and S.J.E.S. L.S. helped with the *in vivo* DT experiments. L. Bruens, L. Bornes, and D.S. helped with the sample preparation of the fluorescence-activated cell sorting (FACS) experiments. L.K. analyzed the single-cell sequencing data. J.M. performed the hydrogel-based assay. D.P. performed the *in vitro* DT cleaved caspase-3 assay. T.S., S.v.d.E., M.v.B., and D.B. operated the FACS. A.M.S. and J.W.M.M. contributed knowledge and reagents. K.C.O. and M.C.H. provided the human organoid line. Experiments were designed with the help of N.V. and H.J.S. J.v.R. together with A.F. designed the experiments and supervised the study. A.F., S.I.J.E., and J.v.R. wrote the manuscript, and the manuscript was approved by all authors.

DECLARATION OF INTERESTS

The authors declare no competing interests.

Received: July 23, 2019
Revised: December 24, 2019
Accepted: February 13, 2020
Published: March 12, 2020

REFERENCES

Al-Hajj, M., Wicha, M.S., Benito-Hernandez, A., Morrison, S.J., and Clarke, M.F. (2003). Prospective identification of tumorigenic breast cancer cells. Proc. Natl. Acad. Sci. U S A *100*, 3983–3988.

Batlle, E., and Clevers, H. (2017). Cancer stem cells revisited. Nat. Med. *23*, 1124–1134.

Beck, B., and Blanpain, C. (2013). Unravelling cancer stem cell potential. Nat. Rev. Cancer *13*, 727–738.

Beerling, E., Seinstra, D., de Wit, E., Kester, L., van der Velden, D., Maynard, C., Schäfer, R., van Diest, P., Voest, E., van Oudenaarden, A., et al. (2016). Plasticity between epithelial and mesenchymal states unlinks EMT from metastasis-enhancing stem cell capacity. Cell Rep. *14*, 2281–2288.

Bonnet, D., and Dick, J.E. (1997). Human acute myeloid leukemia is organized as a hierarchy that originates from a primitive hematopoietic cell. Nat. Med. *3*, 730–737.

Chen, J., Li, Y., Yu, T.S., McKay, R.M., Burns, D.K., Kernie, S.G., and Parada, L.F. (2012). A restricted cell population propagates glioblastoma growth after chemotherapy. Nature *488*, 522–526.

Chen, J., Gong, C., Mao, H., Li, Z., Fang, Z., Chen, Q., Lin, M., Jiang, X., Hu, Y., Wang, W., et al. (2018). E2F1/SP3/STAT6 axis is required for IL-4-induced epithelial-mesenchymal transition of colorectal cancer cells. Int. J. Oncol. *53*, 567–578.

Cortina, C., Turon, G., Stork, D., Hernando-Momblona, X., Sevillano, M., Aguilera, M., Tosi, S., Merlos-Suárez, A., Stephan-Otto Attolini, C., Sancho, E., and Batlle, E. (2017). A genome editing approach to study cancer stem cells in human tumors. EMBO Mol. Med. *9*, 869–879.

de Sousa e Melo, F., Kurtova, A.V., Harnoss, J.M., Kljavin, N., Hoeck, J.D., Hung, J., Anderson, J.E., Storm, E.E., Modrusan, Z., Koeppen, H., et al. (2017). A distinct role for $Lgr5^+$ stem cells in primary and metastatic colon cancer. Nature *543*, 676–680.

de Sousa E Melo, F., and de Sauvage, F.J. (2019). Cellular plasticity in intestinal homeostasis and disease. Cell Stem Cell *24*, 54–64.

Donati, G., and Watt, F.M. (2015). Stem cell heterogeneity and plasticity in epithelia. Cell Stem Cell *16*, 465–476.

Driessens, G., Beck, B., Caauwe, A., Simons, B.D., and Blanpain, C. (2012). Defining the mode of tumour growth by clonal analysis. Nature *488*, 527–530.

Drost, J., van Jaarsveld, R.H., Ponsioen, B., Zimberlin, C., van Boxtel, R., Buijs, A., Sachs, N., Overmeer, R.M., Offerhaus, G.J., Begthel, H., et al. (2015). Sequential cancer mutations in cultured human intestinal stem cells. Nature *521*, 43–47.

Fujii, M., Shimokawa, M., Date, S., Takano, A., Matano, M., Nanki, K., Ohta, Y., Toshimitsu, K., Nakazato, Y., Kawasaki, K., et al. (2016). A colorectal tumor organoid library demonstrates progressive loss of niche factor requirements during tumorigenesis. Cell Stem Cell *18*, 827–838.

Fumagalli, A., Drost, J., Suijkerbuijk, S.J., van Boxtel, R., de Ligt, J., Offerhaus, G.J., Begthel, H., Beerling, E., Tan, E.H., Sansom, O.J., et al. (2017). Genetic dissection of colorectal cancer progression by orthotopic transplantation of engineered cancer organoids. Proc. Natl. Acad. Sci. U S A *114*, E2357–E2364.

Fumagalli, A., Suijkerbuijk, S.J.E., Begthel, H., Beerling, E., Oost, K.C., Snippert, H.J., van Rheenen, J., and Drost, J. (2018). A surgical orthotopic organoid transplantation approach in mice to visualize and study colorectal cancer progression. Nat. Protoc. *13*, 235–247.

Guo, W., Keckesova, Z., Donaher, J.L., Shibue, T., Tischler, V., Reinhardt, F., Itzkovitz, S., Noske, A., Zürrer-Härdi, U., Bell, G., et al. (2012). Slug and Sox9 cooperatively determine the mammary stem cell state. Cell *148*, 1015–1028.

Hanahan, D., and Weinberg, R.A. (2011). Hallmarks of cancer: the next generation. Cell *144*, 646–674.

Hirata, E., Girotti, M.R., Viros, A., Hooper, S., Spencer-Dene, B., Matsuda, M., Larkin, J., Marais, R., and Sahai, E. (2015). Intravital imaging reveals how BRAF inhibition generates drug-tolerant microenvironments with high integrin β1/FAK signaling. Cancer Cell *27*, 574–588.

Knuchel, S., Anderle, P., Werfelli, P., Diamantis, E., and Rüegg, C. (2015). Fibroblast surface-associated FGF-2 promotes contact-dependent colorectal cancer cell migration and invasion through FGFR-SRC signaling and integrin αvβ5-mediated adhesion. Oncotarget *6*, 14300–14317.

Lenos, K.J., Miedema, D.M., Lodestijn, S.C., Nijman, L.E., van den Bosch, T., Romero Ros, X., Lourenço, F.C., Lecca, M.C., van der Heijden, M., van Neerven, S.M., et al. (2018). Stem cell functionality is microenvironmentally defined during tumour expansion and therapy response in colon cancer. Nat. Cell Biol. *20*, 1193–1202.

Mani, S.A., Guo, W., Liao, M.J., Eaton, E.N., Ayyanan, A., Zhou, A.Y., Brooks, M., Reinhard, F., Zhang, C.C., Shipitsin, M., et al. (2008). The epithelial-mesenchymal transition generates cells with properties of stem cells. Cell *133*, 704–715.

McLean, I.W., and Nakane, P.K. (1974). Periodate-lysine-paraformaldehyde fixative. A new fixation for immunoelectron microscopy. J. Histochem. Cytochem. *22*, 1077–1083.

Meacham, C.E., and Morrison, S.J. (2013). Tumour heterogeneity and cancer cell plasticity. Nature *501*, 328–337.

Medema, J.P. (2013). Cancer stem cells: the challenges ahead. Nat. Cell Biol. *15*, 338–344.

Muraro, M.J., Dharmadhikari, G., Grün, D., Groen, N., Dielen, T., Jansen, E., van Gurp, L., Engelse, M.A., Carlotti, F., de Koning, E.J., and van Oudenaarden, A. (2016). A single-cell transcriptome atlas of the human pancreas. Cell Syst. *3*, 385–394.e3.

Nassar, D., and Blanpain, C. (2016). Cancer stem cells: basic concepts and therapeutic implications. Annu. Rev. Pathol. *11*, 47–76.

Oost, K.C., van Voorthuijsen, L., Fumagalli, A., Lindeboom, R.G.H., Sprangers, J., Omerzu, M., Rodriguez-Colman, M.J., Heinz, M.C., Verlaan-Klink, I., Maurice, M.M., et al. (2018). Specific labeling of stem cell activity in human colorectal organoids using an ASCL2-responsive minigene. Cell Rep. *22*, 1600–1614.

Patsialou, A., Bravo-Cordero, J.J., Wang, Y., Entenberg, D., Liu, H., Clarke, M., and Condeelis, J.S. (2013). Intravital multiphoton imaging reveals multicellular streaming as a crucial component of in vivo cell migration in human breast tumors. Intravital *2*, e25294.

Ricci-Vitiani, L., Lombardi, D.G., Pilozzi, E., Biffoni, M., Todaro, M., Peschle, C., and De Maria, R. (2007). Identification and expansion of human colon-cancer-initiating cells. Nature *445*, 111–115.

Ritsma, L., Steller, E.J., Ellenbroek, S.I., Kranenburg, O., Borel Rinkes, I.H., and van Rheenen, J. (2013). Surgical implantation of an abdominal imaging window for intravital microscopy. Nat. Protoc. *8*, 583–594.

Ritsma, L., Ellenbroek, S.I.J., Zomer, A., Snippert, H.J., de Sauvage, F.J., Simons, B.D., Clevers, H., and van Rheenen, J. (2014). Intestinal crypt homeostasis revealed at single-stem-cell level by in vivo live imaging. Nature *507*, 362–365.

Roper, J., Tammela, T., Cetinbas, N.M., Akkad, A., Roghanian, A., Rickelt, S., Almeqdadi, M., Wu, K., Oberli, M.A., Sánchez-Rivera, F.J., et al. (2017). In vivo genome editing and organoid transplantation models of colorectal cancer and metastasis. Nat. Biotechnol. *35*, 569–576.

Sato, T., and Clevers, H. (2013). Growing self-organizing mini-guts from a single intestinal stem cell: mechanism and applications. Science *340*, 1190–1194.

Sato, T., Vries, R.G., Snippert, H.J., van de Wetering, M., Barker, N., Stange, D.E., van Es, J.H., Abo, A., Kujala, P., Peters, P.J., and Clevers, H. (2009). Single Lgr5 stem cells build crypt-villus structures in vitro without a mesenchymal niche. Nature *459*, 262–265.

Sato, T., van Es, J.H., Snippert, H.J., Stange, D.E., Vries, R.G., van den Born, M., Barker, N., Shroyer, N.F., van de Wetering, M., and Clevers, H. (2011). Paneth cells constitute the niche for Lgr5 stem cells in intestinal crypts. Nature *469*, 415–418.

Schepers, A.G., Snippert, H.J., Stange, D.E., van den Born, M., van Es, J.H., van de Wetering, M., and Clevers, H. (2012). Lineage tracing reveals Lgr5+ stem cell activity in mouse intestinal adenomas. Science *337*, 730–735.

Schneider, C.A., Rasband, W.S., and Eliceiri, K.W. (2012). NIH Image to ImageJ: 25 years of Image Analysis. Nat. Methods *9*, 671–675.

Schwitalla, S., Fingerle, A.A., Cammareri, P., Nebelsiek, T., Göktuna, S.I., Ziegler, P.K., Canli, O., Heijmans, J., Huels, D.J., Moreaux, G., et al. (2013). Intestinal tumorigenesis initiated by dedifferentiation and acquisition of stem-cell-like properties. Cell *152*, 25–38.

Serra, D., Mayr, U., Boni, A., Lukonin, I., Rempfler, M., Challet Meylan, L., Stadler, M.B., Strnad, P., Papasaikas, P., Vischi, D., et al. (2019). Self-organization and symmetry breaking in intestinal organoid development. Nature *569*, 66–72.

Shimokawa, M., Ohta, Y., Nishikori, S., Matano, M., Takano, A., Fujii, M., Date, S., Sugimoto, S., Kanai, T., and Sato, T. (2017). Visualization and targeting of LGR5^{+} human colon cancer stem cells. Nature *545*, 187–192.

Snippert, H.J., van der Flier, L.G., Sato, T., van Es, J.H., van den Born, M., Kroon-Veenboer, C., Barker, N., Klein, A.M., van Rheenen, J., Simons, B.D., and Clevers, H. (2010). Intestinal crypt homeostasis results from neutral competition between symmetrically dividing Lgr5 stem cells. Cell *143*, 134–144.

Soteriou, D., and Fuchs, Y. (2018). A matter of life and death: stem cell survival in tissue regeneration and tumour formation. Nat. Rev. Cancer *18*, 187–201.

Stuart, T., Butler, A., Hoffman, P., Hafemeister, C., Papalexi, E., Mauck, W.M., 3rd, Hao, Y., Stoeckius, M., Smibert, P., and Satija, R. (2019). Comprehensive integration of single-cell data. Cell *177*, 1888–1902.e21.

Tauriello, D.V.F., Palomo-Ponce, S., Stork, D., Berenguer-Llergo, A., Badia-Ramentol, J., Iglesias, M., Sevillano, M., Ibiza, S., Cañellas, A., Hernando-Momblona, X., et al. (2018). TGFβ drives immune evasion in genetically reconstituted colon cancer metastasis. Nature *554*, 538–543.

Tetteh, P.W., Basak, O., Farin, H.F., Wiebrands, K., Kretzschmar, K., Begthel, H., van den Born, M., Korving, J., de Sauvage, F., van Es, J.H., et al. (2016). Replacement of lost Lgr5-positive stem cells through plasticity of their enterocyte-lineage daughters. Cell Stem Cell *18*, 203–213.

Tian, H., Biehs, B., Warming, S., Leong, K.G., Rangell, L., Klein, O.D., and de Sauvage, F.J. (2011). A reserve stem cell population in small intestine renders Lgr5-positive cells dispensable. Nature *478*, 255–259.

Todaro, M., Alea, M.P., Di Stefano, A.B., Cammareri, P., Vermeulen, L., Iovino, F., Tripodo, C., Russo, A., Gulotta, G., Medema, J.P., and Stassi, G. (2007). Colon cancer stem cells dictate tumor growth and resist cell death by production of interleukin-4. Cell Stem Cell *1*, 389–402.

Todaro, M., Gaggianesi, M., Catalano, V., Benfante, A., Iovino, F., Biffoni, M., Apuzzo, T., Sperduti, I., Volpe, S., Cocorullo, G., et al. (2014). CD44v6 is a marker of constitutive and reprogrammed cancer stem cells driving colon cancer metastasis. Cell Stem Cell *14*, 342–356.

van de Wetering, M., Francies, H.E., Francis, J.M., Bounova, G., Iorio, F., Pronk, A., van Houdt, W., van Gorp, J., Taylor-Weiner, A., Kester, L., et al. (2015). Prospective derivation of a living organoid biobank of colorectal cancer patients. Cell *161*, 933–945.

van der Bij, G.J., Bögels, M., Otten, M.A., Oosterling, S.J., Kuppen, P.J., Meijer, S., Beelen, R.H., and van Egmond, M. (2010). Experimentally induced liver metastases from colorectal cancer can be prevented by mononuclear phagocyte-mediated monoclonal antibody therapy. J. Hepatol. *53*, 677–685.

van Es, J.H., Sato, T., van de Wetering, M., Lyubimova, A., Yee Nee, A.N., Gregorieff, A., Sasaki, N., Zeinstra, L., van den Born, M., Korving, J., et al. (2012). Dll1+ secretory progenitor cells revert to stem cells upon crypt damage. Nat. Cell Biol. *14*, 1099–1104.

Noordmans, H.J., de Roode, R., Staring, M., and Verdaasdonk, R. (2006). Registration and analysis of in vivo multispectral images for correction of motion and comparison in time. Proc. SPIE *6081*, 608106.

Vermeulen, L., De Sousa E Melo, F., van der Heijden, M., Cameron, K., de Jong, J.H., Borovski, T., Tuynman, J.B., Todaro, M., Merz, C., Rodermond, H., et al. (2010). Wnt activity defines colon cancer stem cells and is regulated by the microenvironment. Nat. Cell Biol. *12*, 468–476.

Ye, X., Tam, W.L., Shibue, T., Kaygusuz, Y., Reinhardt, F., Ng Eaton, E., and Weinberg, R.A. (2015). Distinct EMT programs control normal mammary stem cells and tumour-initiating cells. Nature *525*, 256–260.

Zomer, A., Ellenbroek, S.I., Ritsma, L., Beerling, E., Vrisekoop, N., and Van Rheenen, J. (2013). Intravital imaging of cancer stem cell plasticity in mammary tumors. Stem Cells *31*, 602–606.

STAR★METHODS

KEY RESOURCES TABLE

REAGENT or RESOURCE	SOURCE	IDENTIFIER
Antibodies		
Mouse monoclonal anti-β-catenin	BD Biosciences	Cat# 610154; RRID: AB_397555
Goat polyclonal anti-GFP	Abcam	Cat# ab6673; RRID: AB_305643
Rabbit polyclonal anti-RFP	Rockland	Cat# 600-401-379; RRID: AB_2209751
Mouse monoclonal CD41a, Biotin	Thermo Fisher Scientific	Cat# 13-0411-82; RRID: AB_763484
Mouse monoclonal CD45, Biotin	Thermo Fisher Scientific	Cat# 13-0451-85; RRID: AB_466447
Alexa fluor 488 donkey anti-goat	Abcam	Cat# ab150129; RRID: AB_2687506
Alexa fluor 568 donkey anti-rabbit	Abcam	Cat# ab175470; RRID: AB_2783823
Alexa fluor 647 streptavidin	Thermo Fisher Scientific	Cat# S-21374; RRID: AB_2336066
Chemicals, Peptides, and Recombinant Proteins		
Tamoxifen	Merk	Cat# T5648
Advanced DMEM F/12	Thermo Fisher Scientific	Cat# 12634-010
B27	Thermo Fisher Scientific	Cat# 17504-044
BME	R&D Systems	Cat# 3533-005-02
Buprenorphine	Multidosis-Astfarma	N/A
Collagenase II	Thermo Fisher Scientific	Cat# 17101015
Diptheria Toxin	Merk	Cat# D0564
DNase I	Merk	Cat# 4716728001
Fetal Bovine Serum	Merk	Cat# F7524
FGF	Thermo Fisher Scientific	Cat# PHG0021
HGF	R&D Systems	Cat# 294-HG
Hyaluronidase	Merk	Cat# HX0514-1
IL13	Peprotech	Cat# 214-13
IL4	Peprotech	Cat# 214-14
Matrigel	Corning	Cat# 356231
N-acetylcysteine	Merk	Cat# A9165
Normal Goat Serum	Monosan	Cat# monx10961
Puramatrix Hydrogel	Corning	Cat# 354250
Purified fibronectin	Merk	Cat# FC010
Purified laminin-111	Merk	Cat# L2020
Rat Tail High Concentrated Type I Collagen	Corning	Cat# 354249
Recombinant human Noggin	PeproTech	Cat# 120-10C
Tissue-Tek OCT	Sakura	Cat# 4583
TrypIE	Thermo Fisher Scientific	Cat# 12605-010
Y-27632	Abmole	Cat# M1817
Dapi	Thermo Fisher Scientific	Cat# D1306
Deposited Data		
Single cell mRNA sequencing	This study	GSE143988
Experimental Models: Cell Lines		
Murine_VillinCre-ER^{T2}; APC$^{fl/fl}$; KRAS$^{LSL-G12D/+}$; P53$^{KO/KO}$; R26R-Confetti; Lgr5$^{DTR-eGFP}$	This study	N/A
Human CRC	van de Wetering et al., 2015	p19bT
Experimental Models: Organisms/Strains		
*Mus musculus*_B6.Cg-Tg(Vil1-cre/ERT2)23Syr/J	The Jackson Laboratory	Cat# 020282
Mus musculus _APCtmTno (580S flox)	APB	Cat# 5416

(*Continued on next page*)

Continued		
REAGENT or RESOURCE	SOURCE	IDENTIFIER
Mus musculus _B6.129S4-Kras^{tm4Tyj}/J	The Jackson Laboratory	Cat# 008179
Mus musculus _B6.129S2-Trp53^{tm1Tyj}/J		
Mus musculus _Gt(ROSA) 26Sor$^{tm1(CAG-Brainbow2.1)Cle}$/J	The Jackson Laboratory	Cat# 013731
*Mus musculus*_Lgr5DTR-eGFP	Genentech (Tian et al., 2011)	N/A
Mus musculus _NOD.Cg-Prkdc^{scid} Il2rg^{tm1Wjl}/SzJ	The Jackson Laboratory	Cat# 005557
Software and Algorithms		
FlowJo 10.6.1	BD Biosciences	https://www.flowjo.com/
GraphPad Prism	GraphPad Software	https://www.graphpad.com/scientific-software/prism/
Fiji (ImageJ)	Schneider et al., 2012	https://imagej.nih.gov/ij/
Jvr Jittering Corrector		N/A
Match motion compensation software	Noordmans et al., 2006	N/A
R software	GNU project	https://www.r-project.org/

LEAD CONTACT AND MATERIALS AVAILABILITY

Further information and requests for resources and reagents should be directed to and will be fulfilled by the Lead Contact, Jacco van Rheenen (j.v.rheenen@nki.nl).

All unique/stable reagents generated in this study are available from the Lead Contact with a completed Materials Transfer Agreement.

EXPERIMENTAL MODEL AND SUBJECT DETAILS

Generation of a colorectal cancer genetic mouse model

The VillinCre-ER^{T2} mouse model (The Jackson Laboratory, cat. no. 20282) was combined with the APC$^{fl/fl}$, KRAS$^{LSL\text{-}G12D/+}$, TP53$^{KO/KO}$ mouse model in order to generate a VillinCre-ER^{T2}; APC$^{fl/fl}$; KRAS$^{LSL\text{-}G12D/+}$; TP53$^{KO/KO}$, a Tamoxifen-inducible triple mutant genetic mouse model. The color randomizer R26R-Confetti (gifted by the Clevers laboratory, Hubrecht Institute, NL (Snippert et al., 2010) allele and stem cell fluorescent reporter Lgr5$^{DTR\text{-}eGFP}$ (gifted by De Sauvage laboratory, Genentech, USA (Tian et al., 2011)) were bred in to finally generate the VillinCre-ER^{T2}; APC$^{fl/fl}$; KRAS$^{LSL\text{-}G12D/+}$; TP53$^{KO/KO}$; R26R-Confetti; Lgr5$^{DTR\text{-}eGFP}$ genetic mouse model in a mixed background. In these mice Lgr5^{+} cells (e.g., the stem cells in the intestine) are labeled with membrane-bound eGFP. Activation of the inducible Cre upon administration of Tamoxifen initiates formation of tumors which are restricted to the intestine and promotes the expression of one of the four Confetti colors, thereby specifically color-coding the tumor cells. Since tumors develop throughout the whole intestinal tract, these mice reach the humane endpoint 5 to 6 days after high dose (200mg/kg) Tamoxifen (Merk cat. no. T5648) injection.

Isolation and culturing of mouse organoids

8 weeks old VillinCre-ER^{T2}; APC$^{fl/fl}$; KRAS$^{LSL\text{-}G12D/+}$; TP53$^{KO/KO}$; R26R-Confetti; Lgr5$^{DTR\text{-}eGFP}$ transgenic mice were injected with 200mg/kg Tamoxifen and sacrificed 4 days after induction. Colorectal cancer organoids were isolated from two independent VillinCre-ER^{T2}; APC$^{fl/fl}$; KRAS$^{LSL\text{-}G12D/+}$; TP53$^{KO/KO}$; R26R-Confetti; Lgr5$^{DTR\text{-}eGFP}$ transgenic mice as previously described(Sato et al., 2009). Minimal colorectal cancer organoid culture medium (minimal CRC medium) contained advanced DMEM/F12 medium (adDMEM/F12; Thermo Fisher Scientific, cat. no. 12634-010), B27 2% (Thermo Fisher Scientific, cat. no. 17504-044), N-acetylcysteine 1.25 mM (Merk, cat. no. A9165) and Noggin 1% (PeproTech, cat. no. 120-10C). In order to discriminate between CSCs (Lgr5$^{DTR\text{-}eGFP}$) and Lgr5^{-} tumor cells, RFP-Confetti labeled cells were isolated by flow cytometry and expanded in culture as tumor organoid lines.

Acceptor mice for transplantation experiments

8 – 14 weeks old male and/or females NOD.Cg-Prkdc^{scid} Il2rg^{tm1Wjl}/SzJ (NSG, The Jackson Laboratory cat. no. 005557) mice were used as acceptors for subcutaneous injection, orthotopic transplantation and mesenteric vein injection. All experiments were performed in accordance with the Animal Welfare Committees of the Royal Netherlands Academy of Arts and Sciences and the Netherlands Cancer Institute, the Netherlands. Animals were kept at the Hubrecht animal facility in Utrecht or at the Netherlands Cancer Institute facility in Amsterdam, the Netherlands. For every experimental condition, at least 4 mice were used. No animals were excluded from analyses. When experiments consisted of multiple conditions, either males or females of the same litter were randomly assigned to each group.

Human organoids

The experiments were performed with patient-derived P19bT human organoids (van de Wetering et al., 2015), mutated in TP53, PIK3CA, BRAF, ERBB3, RNF43) constitutively labeled with H2B-mNeonGreen while cancer stem cells specifically express singleTomato-NLS driven by a ASCL2-responsive minigene (STAR, (Oost et al., 2018)).

METHOD DETAILS

Orthotopic transplantation of murine organoids

Orthotopic transplantation of colorectal cancer organoids was performed as previously described (Fumagalli et al., 2017, 2018). In brief, the day before transplantation organoids were collected and dissociated into small cell clumps. About 250,000 cells were plated in 15 μl drops neutralized Rat Tail High Concentrated Type I Collagen (Corning, cat. no. 354249) and let to recover overnight at 37°C, 5% CO_2 in CRC medium containing 100 μM Y-27632 (Abmole, cat. no. M1817). At the day of transplantation, acceptor mice were sedated using isoflurane inhalation anesthesia (~2% isoflurane/O_2 mixture). Before surgery, the mice were treated subcutaneously with a single dose of buprenorphine (Buprecare, Multidosis-Astfarma, 3μg per mouse). The cecum was exposed through a midline abdominal incision and a collagen drop containing tumor cells was surgically transplanted in the caecal submucosa. The tumor growth was monitored weekly by abdominal palpation.

Orthotopic transplantation of human organoids

Transplantations were performed as described above using 1.5 10^5 human organoid-derived cells embedded in Rat Tail High Concentrated Type I Collagen (Corning, cat. no. 354249).

Flow cytometry on organoids

Colorectal cancer organoids were collected and mechanically dissociated with a fire-pointed glass pipet. Subsequently cells were incubated in in TryplE (Thermo Fisher Scientific, cat. no. 12605-010) mix containing 100 μM Y-27632 (Abmole Bioscience, cat. no. M1817), 4μg/ml DNase I (Sigma-Aldrich, cat. no. 10104159001) for 10 minutes at 37°C and spun down. Cells were resuspended in FACS buffer containing 2% B27 (Thermo Fisher Scientific, cat. no. 17504-044), 1.25 mM N-acetylcysteine (Sigma-Aldrich, cat. no. A9165), 1% Noggin (PeproTech, cat. no. 120-10C), 100 μM Y-27632 (Abmole Bioscience, cat. no. M1817), 4μg/ml DNase I (Merk, cat. no.4716728001), 0.01% FCS (Merk, cat. no. F7524) in PBS and filtered through a 100 μm and 35 μm strainers (BD Falcon). DAPI 1ng/ml (Thermo Fisher Scientific, cat. no. D1306) was added right before sorting. Cells were sorted on a FACS AriaII Special Ordered Research Product or a FACS Aria Fusion (BD Biosciences). The sort strategy is illustrated in Figures S1J–S1N. A broad FSC/SSC gate was followed by gates excluding doublets and selection of $DAPI^-$-living cells. RFP-$Confetti^+$ tumor cells were subdivided in (Lgr5)$eGFP^+$ and (Lgr5)$eGFP^-$ using stringent gating. For each experiment, the quality of the sorting was assessed by examination of the sorted cells at a confocal microscope (as in Figure S1O).

Flow cytometry on primary mouse material

Orthotopic colorectal cancer tumors were collected and minced on ice using sterile scalpels. The tumor mass was digested at 37°C for about 1 hour in 5mg/ml Collagenase II brown powder (Thermo Fisher Scientific, cat. no. 17101015) dissolved in advanced DMEM/F12 medium (adDMEM/F12; Thermo Fisher Scientific, cat. no. 12634-010) containing 100 μM Y-27632 (Abmole Bioscience, cat. no. M1817), 4μg/ml DNase I (Merk, cat. no. 4716728001), 20 μg/ml Hyaluronidase (Merk, cat. no. HX0514-1). Digested cell clumps were incubated in TryplE (Thermo Fisher Scientific, cat. no. 12605-010) mix containing 100 μM Y-27632 (Abmole Bioscience, cat. no. M1817), 4μg/ml DNase I (Merk, cat. no. 4716728001) for 15 minutes at 37°C and spun down. The cell pellet was resuspended in FACS buffer (composition described above) and filtered through a 35 μm strainer (BD Falcon). DAPI 1ng/ml (Thermo Fisher Scientific, cat. no. D1306) was added right before sorting.

Blood was collected from mice bearing metastatic CRC either via portal vein puncture or right-ventricle cardiac puncture, 7-10 weeks after orthotopic colorectal cancer organoids transplantation. The red blood cells were depleted by NH_4Cl treatment. The remaining circulating tumor cells and immune cells were spun down (4 minutes 500 RCF at RT). Tumor cells and blood cells were blocked in 80% FACS buffer / normal goat serum (Monosan, monx10961) for 10 minutes on ice before labeling with the following antibodies: biotin-conjugated anti-mouse CD41 clone eBioMWReg30 (Thermo Fisher Scientific, cat. no. 13-0411-82) and anti-mouse CD45 clone 30-F11 (Thermo Fisher Scientific, cat. no. 13-0451-85). Secondary labeling was performed using streptavidin-conjugated AF647 (Thermo Fisher Scientific. no. S-32357). Cells were filtered through a 35 μm strainer (BD Falcon). DAPI 1ng/ml (Thermo Fisher Scientific, cat. no. D1306) was added right before sorting.

Cells were sorted on a FACS AriaII Special Ordered Research Product or a FACS Aria Fusion (BD Biosciences). The sort strategy is illustrated in Figures S1J and S1L and Figures S3A and S3D. A broad FSC/SSC gate was followed by gates excluding doublets and selection of $DAPI^-$-living cells. Afterward, immune cells and megakaryocytes were excluded in a dump channel. RFP-$Confetti^+$ tumor cells were subdivided in Lgr5-$eGFP^+$ and Lgr5-$eGFP^-$ using stringent gating. The quality of the sorting was assessed afterward by examination of the cells at a confocal microscope.

Flow cytometry on human xenografts

Blood was collected from mice bearing human metastatic CRC either via right-ventricle cardiac puncture, 7-10 weeks after orthotopic colorectal cancer organoids transplantation. Samples were prepared and analyzed at the FACS as described above for experiments with the murine CRC model.

H2B-mNeonGreen^{+} tumor cells were subdivided in STAR-NLS-singleTomato^{+} and STAR-NLS-singleTomato^{-} using stringent gating. The quality of the sorting was assessed by post-sort purity check and examination of the cells at a confocal microscope.

Immunohistochemistry and fluorescent imaging

Tissues were fixed periodate-lysine-4% paraformaldehyde (PLP)(McLean and Nakane, 1974) buffer overnight at 4°C, incubated in 30% sucrose overnight at 4°C and embedded in Tissue-Tek (Sakura, cat. no. 4583). Organs were cryosectioned and staining were performed on 20-30 μm sections. The stainings were performed with the following primary antibodies: anti-β-catenin clone 14 (BD Bioscience, cat. no. 610154), anti-GFP (Abcam, cat. no. ab6673), anti-RFP (Rockland, cat. no. 600-401-379). Stained sections were imaged with inverted Leica TCS SP5 and TCS SP8 confocal microscopes (Mannheim, Germany). All images were collected in 12 bit with 25X water immersion (HC FLUOTAR L N.A. 0.95 W VISIR 0.17 FWD 2.4 mm) or 20X dry (HCX IRAPO N.A. 0.70 WD 0.5 mm) objectives.

In vivo time-lapse imaging of primary tumors

About 250,000 VillinCre-ER^{T2}; APC$^{fl/fl}$; KRAS$^{LSL-G12D/+}$; TP53$^{KO/KO}$; RFP-Confetti; Lgr5$^{DTR-eGFP}$ organoid-derived cells were mixed in 100 μl Matrigel (Corning, cat. no. 356231) and injected subcutaneously into the flank of recipient NSG mice. Experiments were performed on 9 NSG mice with organoid lines isolated from two independent VillinCre-ER^{T2}; APC$^{fl/fl}$; KRAS$^{LSL-G12D/+}$; TP53$^{KO/KO}$; R26R-Confetti; Lgr5$^{DTR-eGFP}$ transgenic mice. Specifically, 4 NSG were injected with the organoid line derived from the first GEMM donor, 5 NSG with organoid line derived from the second GEMM donor. Time-laps intravital imaging was performed on tumors of about 125 mm^3. During the entire procedure mice were sedated using isoflurane inhalation anesthesia (1.5% isoflurane/ O_2 mixture). The tumor was surgically exposed, and the mouse was placed in a custom designed imaging box with its head in a facemask constantly delivering anesthesia. The imaging box and the microscope were adjusted at 36.5°C using a climate chamber. Intravital images were acquired with an inverted Leica TCS SP5 or TCS SP8 AOBS two-photon microscope (Mannheim, Germany) with a chameleon Ti:Sapphire pumped Optical Parametric Oscillator (Coherent Inc. Santa Clare, CA, USA). The microscopes are equipped with 2 non-descanned and 2 hybrid detectors: GFP and RFP were simultaneously excited at 940 nm and detected with hybrid detectors. Second harmonic generation (Collagen I stroma) was detected with a non-descanned detector. Images were collected every hour for a period of 4 to 8 hours during which the mouse was kept sedated and alive, constantly hydrated with subcutaneous infusion of glucose and electrolytes (NutriFlex special 70/240, Braun, 100 μl/h). All images were collected in 12 bit and acquired with a 25X water immersion (HC FLUOTAR L N.A. 0.95 W VISIR 0.17 FWD 2.4 mm) objective.

Multi-day imaging of liver metastasis

Lgr5^{-} cancer cells were seeded into the liver parenchyma of acceptor mice either by mesenteric vein injection (of about 400,000 Lgr5^{-} sorted cells) or direct liver parenchyma injection (of about 60,000 Lgr5^{-} sorted cells). For the diphtheria toxin experiment mice were treated every other day with 50 μg/kg Diphtheria Toxin (Merk, cat. no. D0564) administered via intraperitoneal injection every other day. 6-8 days after cells seeding, an abdominal imaging window was applied on the liver of the acceptor mice (Ritsma et al., 2013). Before surgery the mice were treated with a sub-cutaneous injection of buprenorphine (Buprecare, Multidosis-Astfarma, 3mg per mouse). The surgery was performed under aseptic conditions. Animals were sedated with ~2% isoflurane/compressed air mixture. After surgery, the mice were kept at 37°C until fully recovered. For every imaging session, mice were sedated using isoflurane inhalation anesthesia (~1.0% isoflurane/ compressed air mixture). The mice were placed in a custom designed imaging box while kept under constant anesthesia. The imaging box and the microscope were adjusted at 36.5°C using a climate chamber. Between the imaging sessions, mice were let recover in their cage. Intravital images were acquired with an inverted Leica SP8 Dive system (Mannheim, Germany) with a MaiTai eHP DeepSee laser (Spectra-Physics). The imaging areas were retraced in subsequent imaging sessions by storing the stage coordinates, and by visual landmarks such as blood vessels as described in[43]. The microscope is equipped with a 4Tune non-descanned detector configured with 4 hybrid detectors: Lgr5-DTR^{eGFP}, Confetti-RFP were simultaneously excited at 940 nm and detected with non-descanned hybrid detectors, together with second harmonic generation (Collagen I, stroma). All images were collected in 12 bit and acquired with a 25x water immersion objective with a free working distance of 2.40 mm (HC FLUOTAR L 25x/0.95 W VISIR 0.17).

Mesenteric vein injection

FACS-sorted cells were resuspended in 100 μl of sterile PBS and injected in the mesenteric vein of acceptor mice as previously described(van der Bij et al., 2010). Mice were sedated isoflurane inhalation anesthesia (~2% isoflurane/ O_2 mixture). Before surgery, the mice were treated with a sub-cutaneous dose of buprenorphine (Buprecare, Multidosis-Astfarma, 3mg per mouse). After surgery, the mice were kept at 37°C until fully recovered.

Intrahepatic injection

FACS-sorted cells were resuspended in 100 μl of sterile Matrigel (Corning, cat no. 356231) and PBS mix (2.5:1) and injected the liver parenchyma. The surgical procedure was performed under isoflurane inhalation anesthesia (~2% isoflurane/ O_2 mixture). Before surgery, the mice were treated with a sub-cutaneous dose of buprenorphine (Buprecare, Multidosis-Astfarma, 3mg per mouse). After surgery, the mice were kept at 37°C until fully recovered.

Organoid formation assays

$Lgr5^+$ cancer stem cells, $Lgr5^-$ cancer cells from either organoids or primary tumors and $Lgr5^-$ circulating tumor cells were isolated by FACS according to their eGFP expression level as described before. $Lgr5^+$ cancer stem cells, $Lgr5^-$ cancer cells from either organoids or primary tumors were seeded in 20 μl BME (R&D systems, cat. no. 3533-005-02) drops containing 500 cells/drop while $Lgr5^-$ circulating tumor cells were seeded in 20 μl BME drops containing 100 cells/drop. Cells were cultured in CRC medium (see above) containing 100 μM Y-27632 (Abmole Bioscience, cat. no. M1817). Cells were imaged daily with an inverted Leica TCS SP5 confocal microscopes (Mannheim, Germany). All images were collected in 12bit with a 25X water immersion (HC FLUOTAR L N.A. 0.95 W VISIR 0.17 FWD 2.4 mm) or with a 20X dry (HCX IRAPO N.A. 0.70 WD 0.5 mm) objective. Enhancement of plasticity by microenvironmental factors was tested by collecting 10000 viable $Lgr5^-$ single cells via FACS and plating them in BME (R&D systems, cat. no. 3533-005-02) over three wells with a density of 50-300 cells per well of a glass-bottom 384-well plate (Corning, cat. no. 4581). Organoids were supplemented with either minimal culture medium (control), or medium including 75 ng/ml HGF (R&D Systems, cat. No. 294-HG), 10ng/ml FGF-2 (Thermo Fisher Scientific, cat. no. PHG0021), 20ng/ml IL4 (Peprotech, cat. no. 214-14), 100 ng/mL IL13 (Peprotech, cat. no. 214-13). Organoids were imaged live using a Leica-based spinning disk confocal microscope equipped with an Andor Dragonfly system, using a water 25x objective (HC-Fluotar, N.A. 0.95) with the Argon-laser of 488nm and the Diode-laser of 561nm using a 40μm pinhole and Andor sCMOS Zyla 4 2p camera. while kept at 37°C and and 5% CO2 overflow. Organoids were scored at day 0 and after 5 (Figure 4F) or10 days (Figure S4K) of culture for expression of $Lgr5^{eGFP}$.

In vitro Diphtheria Toxin treatment

$Lgr5^+$ cancer stem cells, $Lgr5^-$ cancer cells from either organoids or primary tumorrs were seeded in BME as described above. Diphtheria Toxin (100ng/ml, Merk, cat. no. D0564) or vehicle (demi-water) were added to the CRC culture medium (see above) containing 100 μM Y-27632 (Abmole Bioscience, cat. no. M1817). The medium was refreshed every other day.

In vivo Diphtheria Toxin treatment

50 μg/kg Diphtheria Toxin (Merk, cat. no. D0564) or vehicle (demi-water) were administered via intraperitoneal injection every other day.

Hydrogel assay

Organoid-derived Lgr5+ and Lgr5- were sorted as described above, resuspended in 20% sucrose and mixed with Puramatrix Hydrogel (3mg/ml, Corning, cat. no. 354250), purified fibronectin (0.25mg/ml, Merck, cat. no. FC010), purified laminin-111 (50 μg/ml, Merk, cat. no. L2020) The mixture was plated as a drop and gelation of the hydrogel was initiated by carefully adding advanced DMEM/F12 medium (adDMEM/F12; Thermo Fisher Scientific, cat. no. 12634-010). Hydrogels were allowed to form for 30 min at 37C after which the medium was changed to full organoid growth medium with 100 μM Y-27632 (Abmole Bioscience, cat. no. M1817). Organoid medium was refreshed every two-three days.

Comparison of $Lgr5^+$ and $Lgr5^-$ metastatic efficiency

$Lgr5^+$ and $Lgr5^-$ cells were isolated from 4 different murine primary tumors with stringent gating (sorted strategy described above, Figure S1J and Figure S3A) and post-sort purity was assessed by resorting a fraction of every sorted sample. Only samples above 98% purity used for further *in vivo* experiments. Cells were inoculated via mesenteric vein injection as described above. Every *in vivo* biological replicate included: 1. a recipient mouse injected with 50,000 $Lgr5^-$ cells; 2. a recipient mouse injected with 50,000 $Lgr5^+$ cells; 3. when the purity control is between 98%–100%, a recipient mouse injected with a purity control ($Lgr5^+$ cells), established via the percentage of cells falling out from the stringent $Lgr5^-$ gate during post-sort purity analysis (i.e., cells that might have mistakenly been sorted as $Lgr5^-$). Four weeks after injection the mice were sacrificed and the livers were inspected for presence of metastases under a fluorescence-stereo microscope (Leica). Importantly, none of the mice injected with purity controls showed formation of metastases. Tissues were fixed periodate-lysine-4% paraformaldehyde (PLP) buffer (McLean and Nakane, 1974) overnight at 4°C, incubated in 30% sucrose overnight at 4°C and embedded in Tissue-Tek (Sakura, cat. no. 4583).

Comparison of $STAR^+$ and $STAR^-$ metastatic efficiency

Experiments were performed as described above for murine cancer cells. Human $STAR^+$ and $STAR^-$ cells were isolated by FACS and post-sort purity was assessed by resorting a fraction of every sorted sample. 50,000 $STAR^+$ or $STAR^-$ were injected in the mesenteric vein of acceptor mice and a mouse injected with a purity control ($STAR^+$ cells), established via the percentage of cells falling out from

the stringent STAR⁻ gate during post-sort purity analysis was also included every time that the experiment was performed. Four weeks after injection the mice were sacrificed and the livers were inspected for presence of metastases under a fluorescence-stereo microscope (Leica). Importantly, none of the mice injected with purity controls showed formation of metastases. Tissues were fixed periodate-lysine-4% paraformaldehyde (PLP) buffer (McLean and Nakane, 1974) overnight at 4°C, incubated in 30% sucrose overnight at 4°C and embedded in Tissue-Tek (Sakura, cat. no. 4583).

QUANTIFICATION AND STATISTICAL ANALYSIS

Statistics was performed using GraphPad Prism. Paired or unpaired t test (with Welsh correction) was used when data showed normal distribution (verified with normality tests, provided by GraphPad Prism), whereas Mann-Whitney U test was used for data that did not display normal distribution. Adoption of one statistical test or the other is indicated for each experiment in the figure legend.

Flow cytometry

Data were manually analyzed using FlowJo 10.6.1 (https://www.flowjo.com/).

Fluorescent images (fixed samples)

Images were converted to RGB using Fiji (https://imagej.nih.gov/ij/) and only if necessary, corrected for bleed through, smoothened, cropped, rotated and contrasted linearly. Images were quantified using Fiji.

Intravital imaging of primary tumors

Videos were corrected for XYZ drift using a custom-made software (written in Visual Studio 2010 in the .NET framework for Visual Basics. Codes are available on request from J.v.R.) and if needed additionally with match motion compensation software program to correct for rigid and elastic tissue deformation as extensively described by Noordmans and colleagues (Verdaasdonk, 2006). The Z correction of each time point was done manually and the XY correction was done automatically as follows: all images over time were corrected to the image of the first time point. The correction is based on finding the highest spatial correlation between the images. The spatial correlation for pixels in which the gray value in both images are > 0 were analyzed with the Pearson's correlation coefficient. Then, the imaged that needs to be corrected was moved one pixel to the left, and the correlation was recalculated. If this led to a higher correlation, all pixels in the image were moved one pixel to the left. The correlation was calculated for a move of the corrected image to the left, right, up or down. This procedure was repeated iteratively until the coefficient no long increased. After the full correction, the correctness of the XY correction was visually inspected and manually adjusted if required. To experimentally test the success of the XYZ correction, the position of randomly picked cells were tracked, and if the movement is less than half a cell diameter in 4 hours, we called the correction successful (see examples in Figure 2D).

Images were further processed using Fiji. Frames were converted to RGB and only if necessary, corrected for bleed through with AND/OR function, smoothened, cropped, rotated and contrasted linearly. Migration was quantified manually using Fiji. Statistical analysis was performed in GraphPad Prism using non-parametric Mann-Whitney U test or unpaired t test with Welch's correction.

Intravital imaging of liver metastasis

Images were processed and analyzed using Fiji. Frames were converted to RGB and only if necessary, corrected for bleed through with AND/OR function, smoothened, cropped, rotated and contrasted linearly.

Organoid formation assays

Organoids were counted manually by scoring them using a stereomicroscope. Fluorescent images were processed using Fiji. Frames were converted to RGB and only if necessary, corrected for bleed through with AND/OR function, smoothened, cropped, rotated and contrasted linearly. Lgr5-eGFP expression was scored manually.

Metastasis scoring

Whole livers were sectioned in 50 μm thick slices and metastasis imaged with an inverted Leica TCS SP5 or a Leica TCS SP8 confocal microscope (Mannheim, Germany). All images were collected in 12 bit with 25X water immersion (HC FLUOTAR L N.A. 0.95 W VISIR 0.17 FWD 2.4 mm). Images were converted to RGB using Fiji and only if necessary, corrected for bleed through, smoothened, cropped, rotated and contrasted linearly. Images were quantified using Fiji. Quantification was blinded concerning to the data group allocation. Statistical analysis was performed with GraphPad Prism.

Single-cell mRNA sequencing

Single-cell mRNA sequencing was performed using Sort-seq as described in Muraro et al. (Muraro et al., 2016). Single-cell libraries were sequenced on Illumina NextSeq500 with 75bp paired end reads. Read1 contains the cell barcode and Unique Molecule Identifier, read2 was mapped to the mm10 RefSeq transcriptome using Burrows-Wheeler Aligner with standard parameters. Seurat

(R package Seurat (Stuart et al., 2019)) was used for analyzing the single cell data. Single cell transcriptomes were filtered for cells that had at least 1,000 unique transcripts and subsequently log-normalized to 10,000 transcripts per cell. Clustering was performed on the first 13 principal components and clusters were identified with a resolution of 1. Differential gene expression analysis was performed with the roc test. Cell type identification was performed based on the differentially expressed genes between the clusters.

DATA AND CODE AVAILABILITY

The accession number for the single cell transcriptomic data reported in this paper is GEO: GSE143988.GSE143988

Cell Stem Cell, Volume *26*

Supplemental Information

Plasticity of Lgr5-Negative Cancer Cells

Drives Metastasis in Colorectal Cancer

Arianna Fumagalli, Koen C. Oost, Lennart Kester, Jessica Morgner, Laura Bornes, Lotte Bruens, Lisa Spaargaren, Maria Azkanaz, Tim Schelfhorst, Evelyne Beerling, Maria C. Heinz, Daniel Postrach, Danielle Seinstra, Anieta M. Sieuwerts, John W.M. Martens, Stefan van der Elst, Martijn van Baalen, Debajit Bhowmick, Nienke Vrisekoop, Saskia I.J. Ellenbroek, Saskia J.E. Suijkerbuijk, Hugo J. Snippert, and Jacco van Rheenen

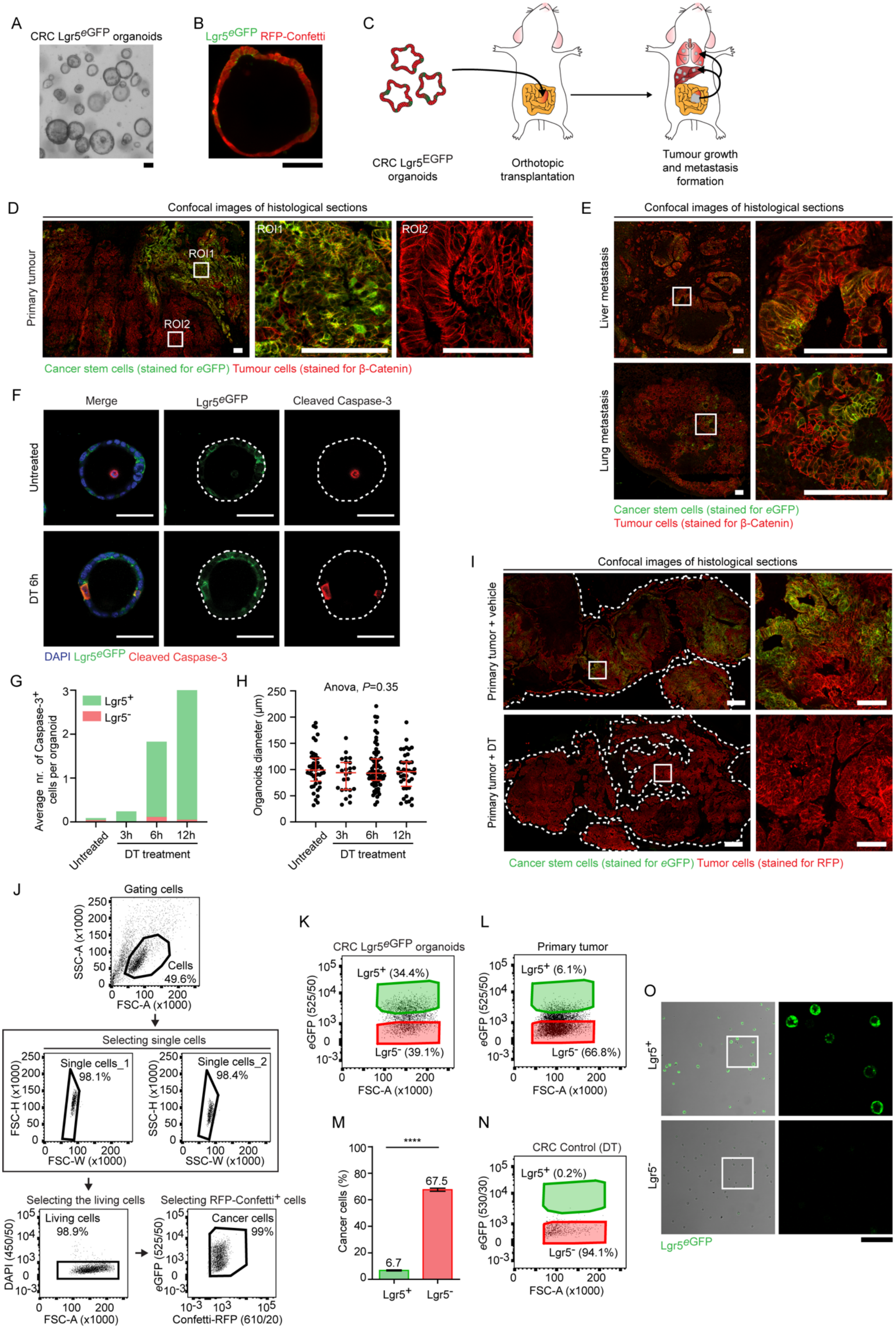
A
CRC Lgr5eGFP organoids
B
Lgr5eGFP RFP-Confetti
C
CRC Lgr5EGFP organoids
Orthotopic transplantation
Tumour growth and metastasis formation
D
Confocal images of histological sections
Primary tumour
ROI1
ROI2
Cancer stem cells (stained for eGFP) Tumour cells (stained for β-Catenin)
E
Confocal images of histological sections
Liver metastasis
Lung metastasis
Cancer stem cells (stained for eGFP)
Tumour cells (stained for β-Catenin)
F
Merge
Lgr5eGFP
Cleaved Caspase-3
Untreated
DT 6h
DAPI Lgr5eGFP Cleaved Caspase-3
G
Average nr. of Caspase-3+ cells per organoid
Lgr5+
Lgr5-
Untreated
3h
6h
12h
DT treatment
H
Anova, P=0.35
Organoids diameter (μm)
Untreated
3h
6h
12h
DT treatment
I
Confocal images of histological sections
Primary tumor + vehicle
Primary tumor + DT
Cancer stem cells (stained for eGFP) Tumor cells (stained for RFP)
J
Gating cells
SSC-A (x1000)
FSC-A (x1000)
Cells 49.6%
Selecting single cells
Single cells_1 98.1%
FSC-H (x1000)
FSC-W (x1000)
Single cells_2 98.4%
SSC-H (x1000)
SSC-W (x1000)
Selecting the living cells
Living cells 98.9%
DAPI (450/50)
FSC-A (x1000)
Selecting RFP-Confetti+ cells
Cancer cells 99%
eGFP (525/50)
Confetti-RFP (610/20)
K
CRC Lgr5eGFP organoids
Lgr5+ (34.4%)
Lgr5- (39.1%)
eGFP (525/50)
FSC-A (x1000)
L
Primary tumor
Lgr5+ (6.1%)
Lgr5- (66.8%)
eGFP (525/50)
FSC-A (x1000)
M
****
Cancer cells (%)
6.7
67.5
Lgr5+
Lgr5-
N
CRC Control (DT)
Lgr5+ (0.2%)
Lgr5- (94.1%)
eGFP (530/30)
FSC-A (x1000)
O
Lgr5+
Lgr5-
Lgr5eGFP

Figure S1 related to Figure 1. (A) Representative example of a CRC organoid culture generated from VillinCre-ER^{T2}; *APC*$^{fl/fl}$; *KRAS*$^{LSL\text{-}G12D/+}$; *P53*$^{KO/KO}$; R26R-Confetti; Lgr5$^{DTR\text{-}eGFP}$ transgenic mice. **(B)** Representative example of a VillinCre-ER^{T2}; *APC*$^{fl/fl}$; *KRAS*$^{LSL\text{-}G12D/+}$; *P53*$^{KO/KO}$; R26R-Confetti; Lgr5$^{DTR\text{-}eGFP}$ endogenously expressing RFP-Confetti, in which CSCs are additionally labelled with membrane-bound *e*GFP (Lgr5$^{DTR\text{-}eGFP}$). **(C)** Experimental setup: VillinCre-ER^{T2}; *APC*$^{fl/fl}$; *KRAS*$^{LSL\text{-}G12D/+}$; *P53*$^{KO/KO}$; R26R-Confetti; Lgr5$^{DTR\text{-}eGFP}$ were orthotopically transplanted in recipient mice. Over time, organoids grow into primary intestinal tumors that spontaneously metastasize to liver and lungs. **(D)** Representative confocal images of orthotopic primary CRC. ROI = region of interest. **(E)** Representative confocal images of spontaneous liver (upper panel) and lung (lower panel) metastases derived from orthotopic CRC. **(F)** Representative confocal images of CRC Lgr5^{eGFP} organoids either untreated or treated with diphtheria toxin (DT) for 6 hours, stained with cleaved-Caspase-3 (red). Scales bars in A-F are all 100 µm. (**G)** Average number of cleaved Caspase-3 positive Lgr5^{+} or Lgr5^{-} cancer cells per CRC Lgr5^{eGFP} organoid, analyzed in different experimental conditions (untreated, DT-treated for either 3, 6 or 12 hours). **(H)** Diameter of CRC Lgr5^{eGFP} organoids included in the cleaved-Caspase-3 analysis. Red lines represent medians ± interquartile range. *P*-value calculated with one-way Anova test. **(I)** Representative examples of primary tumors either treated with vehicle (upper panel) or diphtheria toxin (DT) (lower panel). Dashed lines highlight tumor edges. Scale bars 500 µm (overview), 100 µm (zoom-in). (**J)** FACS strategy enabling the identification and sorting of Lgr5^{+} CSCs and Lgr5^{-} cancer cells in organoids and orthotopic primary tumors. **(K)** Representative example of FACS profile of CRC Lgr5^{eGFP} organoids. **(L)** Representative example of FACS profile of Lgr5^{+} CSCs and Lgr5^{-} cancer cells in primary orthotopic CRC. **(M)** Quantification of Lgr5^{+} CSCs and Lgr5^{-} cancer cells observed in orthotopic CRC by FACS (*n*=8). Data are presented as mean ± SEM, *P*-value ****< 0.0001, calculated with unpaired t-test with Welch's correction. **(N)** Representative example of FACS profile of CRC Lgr5^{eGFP} organoids subjected to selective ablation of Lgr5^{+} CSCs by diphtheria toxin treatment (DT), used to set the Lgr5^{-} gating. **(O)** Representative examples of sorted Lgr5^{+} CSCs and Lgr5^{-} cancer cells. Scales bars are all 100 µm.

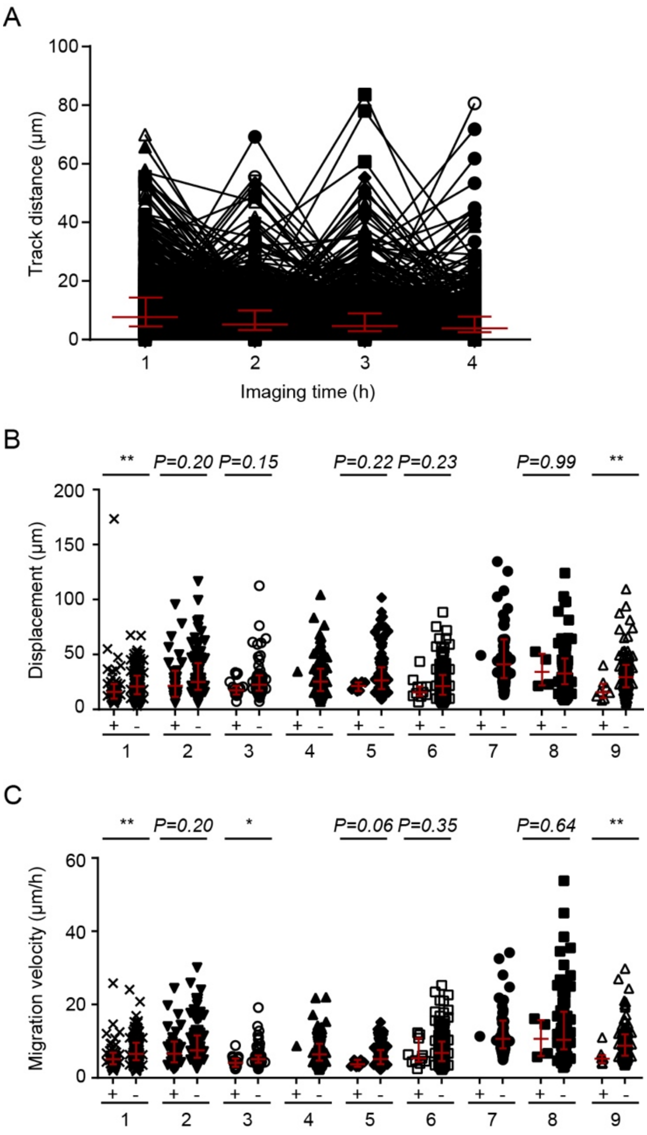

Figure S2 related to Figure 2. (A) Track distance of individual migratory cells followed by intravital imaging over the course of 4 hours. Each line represents an individual cell. Red lines indicate median (per time point) ± interquartile range. **(B-C)** *In vivo* displacement (B) and velocity (C) of escaping $Lgr5^+$ (+) CSCs and $Lgr5^-$ (-) cancer cells imaged in individual mice (n=9). Each data point represents a cell. *P*-value *< 0.05, **< 0.001. Red lines indicate median ± interquartile range. *P*-values calculated with Mann-Whitney U-test.

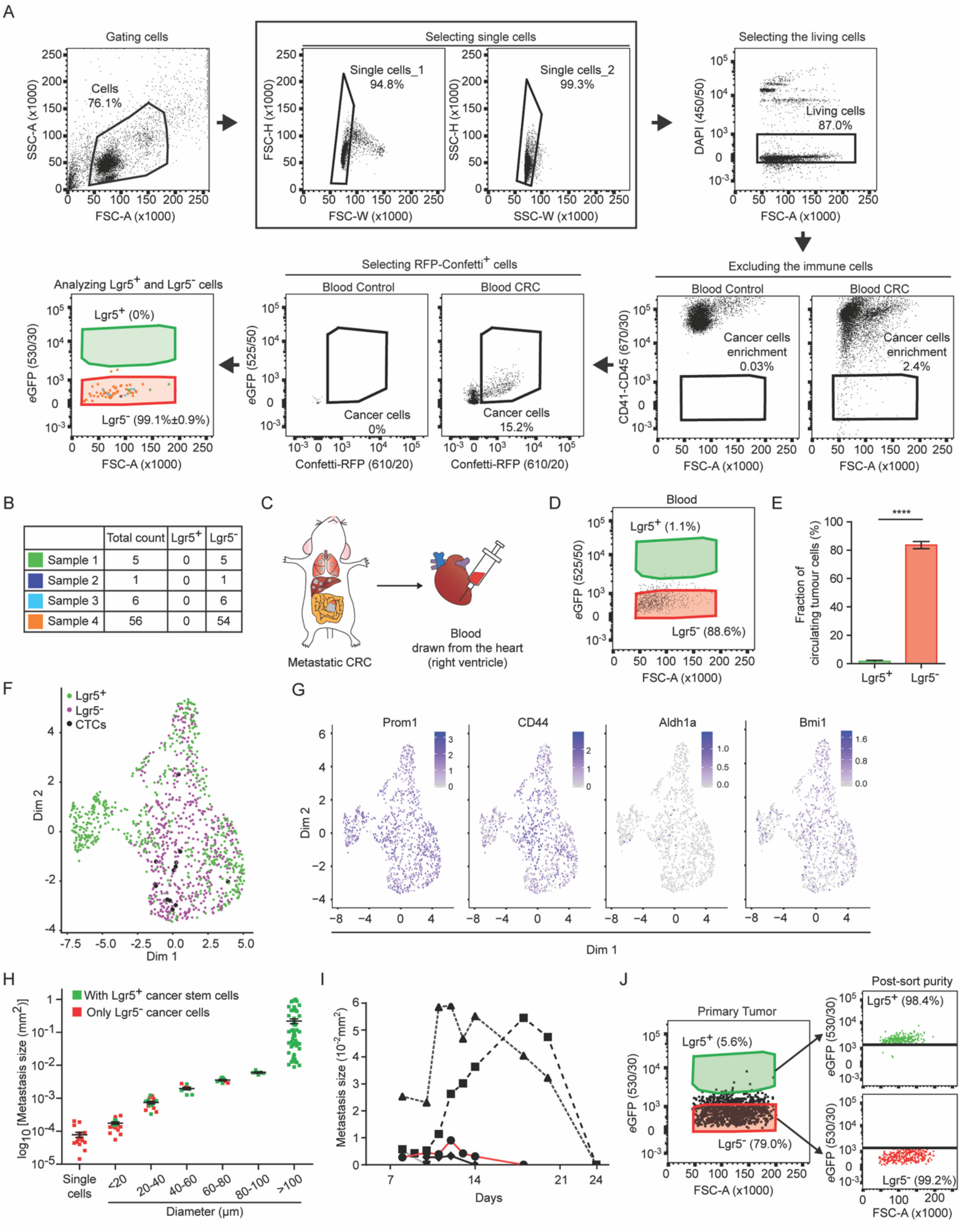

B

	Total count	Lgr5+	Lgr5-
Sample 1	5	0	5
Sample 2	1	0	1
Sample 3	6	0	6
Sample 4	56	0	54

K

Replicate	Lgr5- post-sort purity (%)	Nr. of Lgr5+ cells injected as purity control	Nr. of metastasis in purity control	Nr. of metastasis in Lgr5+ sample	Nr. of metastasis in Lgr5- sample
1	100%	-	0	105	76
2	99.2%	48	0	52	17
3	100%	-	0	90	25
4	100%	-	0	77	24

Figure S3 related to Figure 3. (A) FACS strategy enabling distinctive sorting of circulating $Lgr5^+$ CSCs and $Lgr5^-$ cancer cells. Blood control = blood sample collected from a control NOD.Cg-$Prkdc^{scid}$ $Il2rg^{tm1Wjl}$/SzJ (NSG) mouse. **(B)** Table illustrating the total count of circulating $Lgr5^+$ CSCs and $Lgr5^-$ cancer cells in each blood sample obtained from the portal vein of mice bearing metastatic CRC tumors (see Figure 3A). The colour-coding links individual circulating tumor cells shown in Figure 3B to the corresponding blood sample. **(C)** Experimental setup: A metastatic intestinal tumor model was used to sample blood from the right ventricle of the heart. Blood was analyzed by FACS for the presence of circulating tumor cells. **(D)** Representative example of FACS profile of circulating $Lgr5^+$ CSCs and $Lgr5^-$ cancer cells detected in the systemic blood**. (E)** Quantification of circulating tumor cells found in the systemic blood (n=5). Data are presented as mean ± SEM, P-value ****< 0.0001, calculated with unpaired t-test with Welch's correction. **(F)** tSNE plot representing unbiased clustering of $Lgr5^+$ (green), $Lgr5^-$ (purple) in primary tumor, liver metastasis and circulating tumor cells (CTCs, black). **(G)** tSNE plots showing expression level of selected cancer stem cell marker genes in $Lgr5^+$, $Lgr5^-$ cancer cells of primary tumor, liver metastasis and circulating tumor cells clustered in (F). **(H)** Analysis of spontaneous liver metastases found in mice bearing orthotopic CRC tumors. Metastases are grouped per diameter range and plotted according to size (i.e. area represented in mm^2). Each individual point represents a metastatic lesion (n=132). The colour-coding identifies metastases composed of only $Lgr5^-$ cancer cells (red) and metastases containing $Lgr5^+$ CSCs (green). Black lines represent means ± SEM. **(I)** Diphtheria toxin (DT) treated metastasis plotted by their actual size (μm^2), followed over time by intravital imaging. The red line in represents the example shown in Fig. 3G. **(J-K)** Post-sort purity analysis on $Lgr5^+$ and $Lgr5^-$ sorted populations. (K) $Lgr5^-$ post-sort purity controls calculated in each experiment (n=4) that were performed for the analysis shown in Figure 3J to 3K. The table illustrates the equivalent number of $Lgr5^+$ cells injected in the purity control acceptor mouse and the number of metastases observed in purity control, $Lgr5^+$ and $Lgr5^-$ injected mice in each experimental replicate (n=4). Red highlights the example depicted in (J).

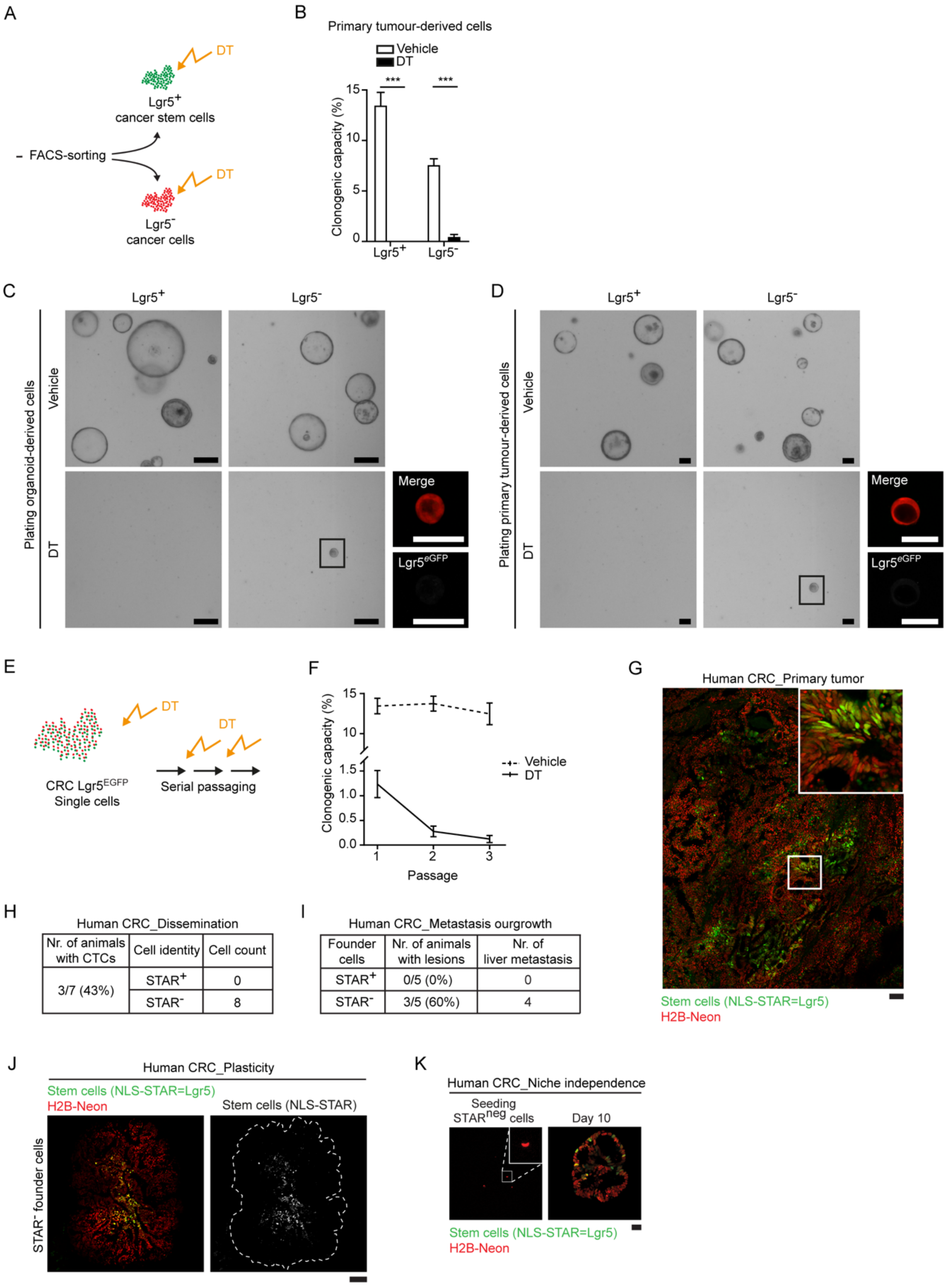
A
DT
Lgr5+ cancer stem cells
FACS-sorting
DT
Lgr5- cancer cells
B
Primary tumour-derived cells
Vehicle
DT
Clonogenic capacity (%)
15
10
5
0
***
***
Lgr5+
Lgr5-
C
Lgr5+
Lgr5-
Plating organoid-derived cells
Vehicle
DT
Merge
Lgr5eGFP
D
Lgr5+
Lgr5-
Plating primary tumour-derived cells
Vehicle
DT
Merge
Lgr5eGFP
E
DT
DT
CRC Lgr5EGFP Single cells
Serial passaging
F
Clonogenic capacity (%)
15
10
1.5
1.0
0.5
0.0
1
2
3
Passage
Vehicle
DT
G
Human CRC_Primary tumor
Stem cells (NLS-STAR=Lgr5)
H2B-Neon
H
Human CRC_Dissemination
Nr. of animals with CTCs | Cell identity | Cell count
3/7 (43%) | STAR+ | 0
STAR- | 8
I
Human CRC_Metastasis ourgrowth
Founder cells | Nr. of animals with lesions | Nr. of liver metastasis
STAR+ | 0/5 (0%) | 0
STAR- | 3/5 (60%) | 4
J
Human CRC_Plasticity
Stem cells (NLS-STAR=Lgr5)
H2B-Neon
Stem cells (NLS-STAR)
STAR- founder cells
K
Human CRC_Niche independence
Seeding STARneg cells
Day 10
Stem cells (NLS-STAR=Lgr5)
H2B-Neon

Figure S4 related to Figure 4. (A-B) Clonogenicity assay of sorted $Lgr5^+$ CSCs and $Lgr5^-$ cancer cells derived from colorectal orthotopic primary tumors. Single cells were subjected to either vehicle or diphtheria toxin (DT) treatment (*n*=3 independent experiments). Data were collected 6 days after plating. Values are presented as mean ± SEM, *P*-value ***< 0.0001, calculated with unpaired t-test with Welch's correction. **(C-D)** Representative example of organoid formation assay of sorted $Lgr5^+$ CSCs and $Lgr5^-$ cancer cells derived from either CRC organoid cultures (C) and orthotopic colorectal primary tumors 6 days after plating (D). Single cells were subjected to either vehicle or diphtheria toxin (DT) treatment. Scale bars, 100 µm. **(E-F)** Clonogenicity assay of CRC $Lgr5^{eGFP}$ organoid-derived cells. Single cells underwent serial passaging (every 6 days) while kept under constant vehicle or diphtheria toxin (DT) treatment (*n*=3 independent experiments). **(G)** Representative confocal image of a human orthotopic primary tumor (mutated in P53, PIK3CA, BRAF, ERBB3, RNF43). DsRed-expressing $Lgr5^+$ cells (using an ASCL2-responsive minigene (STAR) that labels $Lgr5^+$ CSCs with DsRed, Oost et al., 2018) are shown with a green lookup table for consistency with the images of our mouse model. All cancer cells express nuclear mNeon (red lookup table). **(H)** Total count of circulating $STAR^+$ CSCs and $STAR^-$ cancer cells in blood samples obtained from mice bearing human metastatic CRC tumors. **(I)** Number of metastatic foci derived from mesenteric vein injection of $STAR^+$ CSCs or $STAR^-$ cancer cells (*n*=5 biological replicates). **(J)** Representative confocal images of metastases generated from human $Lgr5^-$ cancer cells. **(K)** Representative example of human organoid formation assay of sorted $STAR^-$ cancer cells and cultured in minimum CRC medium (i.e. not containing stem cell-inducing growth factors). Scale bars, 100 µm.
